# Neuronal STAT3/HIF-1α/PTRF axis-mediated bioenergetic disturbance exacerbates cerebral ischemia-reperfusion injury via PLA2G4A

**DOI:** 10.7150/thno.71029

**Published:** 2022-04-04

**Authors:** Weili Jin, Jixing Zhao, Eryan Yang, Yunfei Wang, Qixue Wang, Ye Wu, Fei Tong, Yanli Tan, Junhu Zhou, Chunsheng Kang

**Affiliations:** 1 Department of Neurosurgery, Tianjin Medical University General Hospital, Tianjin 300052, China.; 2 Tianjin Neurological Institute, Key Laboratory of Post-neurotrauma Neuro-repair and Regeneration in Central Nervous System, Ministry of Education, Tianjin City, Tianjin 300052, China.; 3 Department of Pathology, Affiliated Hospital of Hebei University, Baoding 071000, China.

**Keywords:** polymerase I and transcript release factor (PTRF), cerebral ischemia-reperfusion (I/R) injury, lipid metabolism, mitochondrial bioenergetics, oxidative damage

## Abstract

Ischemic stroke is an acute and severe neurological disease with high mortality and disability rates worldwide. Polymerase I and transcript release factor (PTRF) plays a pivotal role in regulating cellular senescence, glucose intolerance, lipid metabolism, and mitochondrial bioenergetics, but its mechanism, characteristics, and functions in neuronal cells following the cerebral ischemia-reperfusion (I/R) injury remain to be determined.

**Methods:** Transcription factor motif analysis, chromatin immunoprecipitation (ChIP), luciferase and co-Immunoprecipitation (co-IP) assays were performed to investigate the mechanisms of PTRF in neuronal cells after I/R injury. Lentiviral-sgRNA against *PTRF* gene was introduced to HT22 cells, and adeno-associated virus (AAV) encoding a human synapsin (hSyn) promoter-driven construct was transduced a short hairpin RNA (shRNA) against PTRF mRNA in primary neuronal cells and the cortex of the cerebral I/R mice for investigating the role of PTRF in neuronal damage and PLA2G4A change induced by the cerebral I/R injury.

**Results:** Here, we reported that neuronal PTRF was remarkably increased in the cerebral penumbra after I/R injury, and HIF-1α and STAT3 regulated the I/R-dependent expression of PTRF via binding to its promoter in neuronal cells. Moreover, overexpression of neuronal PTRF enhanced the activity and stability of PLA2G4A by decreasing its proteasome-mediated degradation pathway. Subsequently, PTRF promoted reprogramming of lipid metabolism and altered mitochondrial bioenergetics, which could lead to oxidative damage, involving autophagy, lipid peroxidation, and ferroptosis via PLA2G4A in neuronal cells. Furthermore, inhibition of neuronal PTRF/PLA2G4A-axis markedly reduced the neurological deficits, cerebral infarct volumes, and mortality rates in the mice following cerebral I/R injury.

**Conclusion:** Our results thus identify that the STAT3/HIF-1α/PTRF-axis in neurons, aggravating cerebral I/R injury by regulating the activity and stability of PLA2G4A, might be a novel therapeutic target for ischemic stroke.

## Introduction

Ischemic stroke results in devastating brain damage and severe neurological deficits due to the sudden cessation of cerebral blood flow to the brain 1, 2, and it is a leading cause of death and long-lasting physical disability worldwide 3. Current clinical interventions focus on the restoration of blood flow to rescue the ischemic penumbra, which surrounds an irreversibly damaged core, suffers a hypoperfusion, and remains potentially salvageable with a limited time window before stroke-induced cellular energy failure and redox dyshomeostasis result in neuronal cell death 1, 2. Notably, once the blood flow is reestablished, a condition called ischemia-reperfusion (I/R) triggers a rapid cascade of neuropathological events, including but not limited to disturbances in cellular bioenergetics and redox homeostasis, which play multifaceted roles in further enhancing brain insults due to the excessive production of reactive oxygen species (ROS) inducing cell death through autophagy, lipid peroxidation and ferroptosis 2, 4, 5. Notwithstanding disturbances in neuronal bioenergetics and redox homeostasis are common neuropathological characteristics of cerebral I/R injury 4, 6, it remains poorly understood their underlying mechanistic and their potential as druggable targets to prevent cerebral I/R injury.

Polymerase I and transcript release factor (PTRF), alternatively known as cavin-1, is a cytoplasmic protein essentially involved in the formation and function of caveola 7. Abundant evidence has suggested that PTRF might possibly be an important candidate for participating in bioenergetics such as lipid reprogramming and mitochondrial bioenergetics in congenital generalized lipodystrophies (CGL) and glioblastoma 8-10. However, little is known about these functions of neuronal PTRF after cerebral I/R injury.

Under hypoxia or ischemia, the hypoxia-inducible transcription factor 1α (HIF-1α) acts as the key mediator in promoting transcription of several genes that have been tightly linked with metabolic networks and redox homeostasis 11-13. Furthermore, signal transducer and activator of transcription 3 (STAT3) are mainly considered to be a direct transcription factor that regulates a large number of genes involved in mitochondrial respiration, ROS production, and autophagy 14, 15. Previous studies have demonstrated that STAT3 promotes the expression of HIF-1α during the ischemic stroke 16. Therefore, we hypothesized that HIF-1α and STAT3 might participate in PTRF-mediated cell signaling events in diverse cellular metabolisms.

PLA2G4A/cPLA2 (phospholipase A2, group IVA [cytosolic, calcium-dependent]) is a crucial regulator of lipid metabolism reprogramming and facilitates mitochondrial bioenergetics in the activated human platelets 17. Our previous study suggests that overexpression of PTRF remodels phospholipid metabolism by stabilizing PLA2G4A in glioblastoma 9. Mechanistically, PLA2G4A cleaves the fatty acyl linkage at the sn-2 position of the glycerol backbone of phosphatidylcholine (PC), liberating lysophosphatidylcholine (LPC) and free fatty acids (FFA) like arachidonic acid 4, 18, leading to a pathological imbalance between PC and LPC ratio as well as ROS overproduction-induced fatal brain damage after cerebral I/R injury 10. Emerging evidence suggests that the expression and activity of PLA2G4A render neuronal cells sensitive to autophagy, and increase lipid peroxidation and ferroptosis following I/R-induced ROS generation 19-22. Importantly, both activity and expression levels of PLA2G4A were increased in neurodegenerative diseases, traumatic brain injury (TBI) and ischemic stroke, and implicated in neuronal cell death 21, making it an attractive target for therapeutic intervention.

In this study, we investigated that expression of PTRF was upregulated in both *in vitro* and *in vivo* neuronal cells following cerebral I/R injury. In addition, we showed that HIF-1α and STAT3 could regulate the I/R-dependent expression of neuronal PTRF via binding to its promoter *in vitro.* Moreover, the neuronal STAT3/HIF-1α/PTRF-axis enhanced the activity and stability of PLA2G4A modulating lipid metabolism reprogramming and mitochondrial bioenergetics, resulting in the increased ROS production to promote autophagy, lipid peroxidation, and ferroptosis. We also demonstrated that neuronal PTRF knockdown (KD) in the mice reduced cerebral I/R injury and improved outcomes via PLA2G4A.

## Materials and methods

### Data collection

Transcriptome data of cerebral I/R injury were downloaded from the GEO database (https://www.ncbi.nlm.nih.gov/geo/), and the dataset of GSE58720 was used in this study.

### Mice

All mice were housed in a pathogen-free facility on a 12-h light/dark cycle and allowed *ad libitum* access to food and water. *In vivo* experiments were performed on 8-10-week-old male C57BL/6 mice. All animal studies were conducted according to the ethical guidelines of experimental animal use and care and approved by the Committee on Animal Ethical and Welfare Committee (AEWC) of Tianjin Medical University General Hospital, China (Approval Number: IRB2020-DW-15).

### MCAO model and harvest of the brain

The middle cerebral artery occlusion (MCAO) model was established through the intraluminal filament method, as previously described 23. Cerebral ischemia was induced in mice for 45 min, and the occluding filament (Doccol Corporation) was subsequently withdrawn for reperfusion. Mice were excluded if the mean cortical blood flow (CBF) was > 20% of pre-ischemic baseline or < 80% of pre-ischemic baseline after reperfusion within 10 min. The mice were kept on a heating plate to maintain their temperature at 37 °C until they regained full consciousness. Standard methods for removing red blood cells and fixing tissues often involve transcardial perfusion, such as brain-targeted perfusion (via the left ventricle) or lung-targeted perfusion (via the right ventricle) 24. Moreover, the mice were sacrificed and perfused, and brain tissues were isolated as previously described 23, 25. In brief, the mice were anesthetized, transcardially perfused (via the left ventricle) with ice-cold PBS (Solarbio, P1010), and sacrificed by cervical dislocation. Next, we removed the head completely from the carcass. Subsequently, small scissors were used to insert the bottom blade into the foramen magnum, and cut directly up and through the midline of the calvaria. Finally, forceps were used to reflect back both halves of the calvaria exposing the brain, and gently pinch any connective tissues that prevent the brain from falling from the skull.

### Flow cytometry

Flow cytometry assay was conducted as our previous study described 23. Briefly, 1 mg/mL collagenase and 0.1 mg/mL DNase I were used to dissociate the ipsilateral cortical tissues in ischemic penumbra into a single-cell suspension according to the manufacturer's instructions. Subsequently, cells were stained with indicated antibodies in 100 μL PBS solution for 30 min at 4 °C. The indicated antibodies used in this study are listed in Table S1. Cell acquisition was performed immediately using the FACSDiva software on a flow cytometer (BD FACSCanto II), and Flowjo 10.6.2 was used to analyze.

### Isolation and culture of primary cells and cell culture

Mouse primary neuronal cells collected from the cerebral cortices of embryos (embryonic day 15) from 10-12-week-old pregnant C57BL/6 mice were isolated and cultured, as described in our previous study 23. In brief, primary neuronal cells were seeded in 6-well plates, or glass-bottom imaging dishes coated with poly-D-lysine (Solarbio, P2100) at a density of 10^5^ cells/mL, and maintained in serum-free neurobasal (Gibco, 10888022) with 1% Glutamax (Gibco, 35050061) and 1% B27 supplement (Gibco, 17504044) at 37 °C in a 5% CO_2_ incubator. Cytosine arabinoside (Sigma, V900339) was added to prevent non-neuronal proliferation after 3 days in culture. Half of the medium was replaced every three days.

For the collection of primary astrocytes, cerebrums of neonatal C57BL/6 mice (1 day) were dissected. Trypsin (0.125%, Gibco, 25200-072) and DNase I (10 U/mL, Solarbio, D8071) were used to homogenize the dissected tissues at 37 °C for 30 min. Primary astrocytes were seeded in poly-L-lysine (Solarbio, P2100) coated flasks at a density of 10^6^ cells/mL and incubated at 37 °C in humidified 5% CO_2_ chamber. The complete culture medium for primary astrocytes was DMEM/F12 medium (Gibco, 10565-018) with 10% FBS and 1% penicillin/streptomycin.

HT22 murine hippocampal cells and BV2 cells were plated in dishes uncoated and cultured at 37 °C with 5% CO_2_ in DMEM (Gibco, 11965-092) with 10% FBS and 1% penicillin/streptomycin. All cell lines used in this study were no more than 25 times.

### Oxygen and glucose deprivation/reoxygenation (OGD/R)

OGD/R experiments were used for the *in vitro* I/R injury model and performed as described in previous study 23, 26. Briefly, HT22 cells and BV2 cells were exposed for 4 h to 1% oxygen in glucose-free DMEM (Gibco, 11966-025) at 37 °C for OGD. The O_2_ levels in the culture medium are equilibrated to 1% hypoxia within 2 h in the cell culture plates under hypoxia 27. To ensure the hypoxic effect, sufficient glucose-free DMEM was added into a dish for 4 h in a hypoxia workstation before OGD. Moreover, the operation of OGD was performed in a hypoxia workstation to eliminate exposure to oxygen in this study. After this period, HT22 cells were incubated in a complete culture medium and saturated with a humidified atmosphere of 5% CO_2_ and 95% air for 8 h reoxygenation before harvest. Control cells were subjected to the same washing and medium changes but always maintained in complete culture medium under conditions of 5% CO_2_ and 95% air at 37 °C. Primary astrocytes and primary neuronal cells were subjected to OGD for 2 h, and then reoxygenation for 10 h.

### RNA extraction and quantitative real-time polymerase chain reaction (qRT-PCR)

Total RNA was extracted from either cultured cells or tissues using TRIzol (Invitrogen, 15596-026). PrimeScript RT reagent kit (TaKaRa, RR037A) was used to reverse total RNA into cDNA, according to the manufacturer's instructions. All gene expression analyses were performed by SYBR Green Master Mix (Applied Biosystems, 4368708) and normalized to β-actin using the 2^-ΔΔCt^ method. PCR primers were designed using a primer designing tool (http://www.ncbi.nlm.nih.gov/tools/primer-blast/) and are listed in Table S2.

### Western blot assay

Protein extraction and immunoblot analysis were performed according to the manufacturer's instructions. The primary antibodies used in this study are listed in Table S1. Appropriate secondary antibodies were HRP-conjugated (Immunoway, RS0002; RS0001; 1:10,000 dilution).

### Immunofluorescence assay

Immunofluorescence (IF) assays were conducted as described in our previous study 23. Briefly, the mice were transcardially perfused with 4% paraformaldehyde (PFA, Solarbio, P1110), and the brains were isolated and post-fixed in 4% PFA for 24 h. HT22 cells, BV2, primary astrocytes or primary neuronal cells were plated on glass-bottom imaging dishes and fixed with 4% PFA at room temperature for 20 min. The brain sections or cells were incubated overnight at 4 °C with indicated primary antibodies (Table S1) in primary antibody diluent. On the next day, the sections or cells were washed three times in PBS and incubated for 1 h at room temperature with the corresponding secondary antibody (Table S1). DNA was stained with 4ʹ, 6-diamidino-2-phenylindole (DAPI; Sigma, F6057) for 10 min at room temperature. Hypoxic regions were detected using a novel hypoxia marker, pimonidazole (Hypoxyprobe-1, MCE, HY-105129A) as described previously 12. Images were obtained using an Olympus FluoView 1200 confocal microscope (Olympus) and analyzed with Olympus Instruments Software. The experimenter that reviewed and analyzed the stained slides was blinded to the groups.

### Cell transfection and lentiviral transduction

The siRNAs and plasmids were obtained from Shanghai Integrated Biotech Solutions Co. Ltd. (Shanghai, China). HT22 cells were transfected with siRNAs, or plasmids using Lipofectamine 3000 (Invitrogen, L3000-015), following manufacturer's instructions. The sequences of siRNAs against specific targets are summarized in Table S3. HT22 cells were subsequently transfected with lentiviruses carrying sgRNAs designed against *PTRF*. After 48 h, expression of PTRF was confirmed by Western blot. Lentiviruses expressing PTRF, Cas9, and sgRNAs targeting *PTRF* were prepared by Genechem.

### Drug administration

Echinomycin (MCE, 512-64-1) and S3I-201 (MCE, 501919-59-1) were dissolved in dimethylsulfoxide (DMSO, Fisher Scientific, 85190) for *in vitro* and *in vivo* experiments. Mice were intraperitoneally injected with indicated concentrations of echinomycin and S3I-201 at the onset of MCAO, and the indicated concentrations of echinomycin and S3I-201 were added to the primary neuronal cells at the onset of OGD. Then, the medium was replaced with a complete medium containing the aforementioned compounds for 10 h-reoxygenation. To determine a posttranscriptional regulatory interaction between PTRF and PLA2G4A in neuron after ischemic stroke, the indicated concentrations of cycloheximide (CHX, 0.100 mM, Sigma-Aldrich, 508739), MG132 (0.010 mM, Selleck, S2619), and chloroquine (CQ, 0.025 mM, Sigma-Aldrich, C6628) were added to HT22 cells at the onset of reoxygenation. For primary neuronal cells, the indicated concentrations of CHX (0.050 mM), MG132 (0.005 mM), and CQ (0.010 mM) were used. Control cells were given an equal medium and drugs.

For *in vitro* treatments, AACOCF3 (arachidonyl trifluoromethyl ketone), a PLA2G4A inhibitor (Cayman Chemical, 62120), was prepared in DMSO (1 mM or 5 mM) and added to the medium at the final concentration of 0.010 mM or 0.050 mM in primary neuronal cells and HT22 cells. The corresponding volume of DMSO was used as a vehicle. To determine the effects of the PLA2G4A inhibitor (AACOCF3) on the cerebral I/R injury, mice were intraperitoneally injected with AACOCF3 9, 21, which was dissolved in DMSO and diluted to 4 mM concentration in saline before injection. AACOCF3 solution (25 mg/kg body weight) was intraperitoneally administered into mice at 1 h pre-, immediately and 3 h post-MCAO surgery. For longer study, mice were injected three times as mentioned here on the day of surgery followed by once a day on day 2 and 3 and then once in every alternate day till day 15.

### HIF-1α DNA binding activity

HIF-1α DNA binding activity assay was performed using HIF-1α Transcription Factor Assay Kit (Abcam, ab133104) as described in previous study 28. We collected treated primary neuronal cells and extracted their nucleoprotein by using Nuclear and Cytoplasmic Protein Extraction Kit (Beyotime, P0027). Samples were added to the wells of transcription factor HIF-1α plate and subsequently incubated overnight at 4 °C without agitation. Diluted HIF-1α primary antibody was added to each well and incubated in the wells for 1 h at room temperature. Next, diluted goat anti-rabbit HRP conjugate was added to each well, and incubated in the wells for 1 h at room temperature. HIF-1α DNA binding activity levels were measured at 450 nm using a microplate reader, following adding the stop solution.

### Chromatin immunoprecipitation (ChIP) assay

ChIP assay was performed using the EZ-Magna ChIP Assay Kit (Millipore, 17-10086) as described previously 29. Briefly, 1×10^7^ cells were cross-linked with 1% PFA/PBS for 10 min at room temperature, and then the unreacted PFA was quenched with 10 ×glycine. Subsequently, samples were sonicated in lysis buffer to obtain 200-1,000 bp DNA fragments to be immunoprecipitated with 5 μg of rabbit HIF-1α, mouse STAT3 or rabbit IgG antibodies (Table S1). The primer sequences specific to the promoter region of *PTRF* gene are listed in Table S4.

### Luciferase reporter assay

To assess *PTRF* gene promotor activity, HT22 cells were seeded into 96-well plates and were co-transfected with PTRF luciferase reporter plasmid, including a tandem repeat of the PTRF transcriptional response element and the *Renilla* control reporter serving as an internal control. After 48 h, cells were lysed, and the enzymatic activity of luciferase and *Renilla* were measured using the Dual-Luciferase Assay kit (Promega, E1910), according to the manufacturer's protocol.

### Co-Immunoprecipitation (Co-IP) assay

Co-IP experiments were performed as described in our previous study 29. Briefly, HT22 cells were harvested and lysed using IP lysis buffer, and then 5 μg of anti-STAT3 or anti-CBP antibody were used to incubate with cell lysates overnight at 4 °C. Samples were incubated with protein A agarose beads with rotation for 3 h at 4 °C. After incubation, immune complexes were isolated by centrifugation and washed three times with lysis buffer. Subsequently, protein complexes were incubated for 5 min at 95 °C after dissolving in the electrophoresis sample buffer. The co-IP was subjected to Western blot for further analysis using specific primary antibodies (Table S1).

### Intracerebral injection of adeno-associated virus (AAV) and adenoviral transduction *in vitro*

According to the previous reports, the ischemic penumbra was mainly located in the middle cortex 30. To genetically downregulate PTRF in the mouse neuronal cells of the ischemic penumbra, AAV-hSyn-sh-PTRF and AAV-hSyn-sh-scramble provided by Shanghai Integrated Biotech Solutions Co. Ltd, were intracerebrally injected at multiple points in the cortex with a microsyringe (5 μL, Hamilton, Martinsried, Germany). Mice were anesthetized, and the right cortex was injected with 2 μL of AAV-hSyn-sh-PTRF or AAV-hSyn-sh-scramble (5 × 10^12^ GC/mL), and then were housed for 2 months before MCAO surgery. Furthermore, AAV-mediated PTRF KD was confirmed by Western blot in the cerebrum. For PTRF KD in primary neuronal cells, after 2 days in culture, the cultured neuronal cells were transduced with AAV-hSyn-sh-PTRF or AAV-hSyn-sh-scramble at a multiplicity of infection (MOI) of 10 for 24 h. Subsequently, KD efficiency in primary neuronal cells was assessed 4 days after transduction by Western blot.

### Seahorse XFe Extracellular Flux Analysis (Mito Stress Test)

Seahorse XFe Extracellular Flux Analysis was conducted as previous study described 9, 26. HT22 cells were seeded into Seahorse XF24 cell culture plate (Agilent, 102342-100) at 3,000 cells per well and allowed to adhere overnight. Primary neuronal cells were isolated, counted and diluted to a density of 10^5^ cells/mL. Subsequently, add 1 mL to each well in a Seahorse XF24 tissue culture plate (Agilent, 100777-004), and primary neuronal cells were cultured for 10 days. Oxygen consumption rate (OCR) was measured using Seahorse XF Cell Mito Stress Test Kit (Agilent, 103015-100) and the extracellular flux analyzer XF24 (Seahorse Bioscience Inc.), according to the manufacturer's protocol. Before measurement, the regular cell culture medium was replaced with the XF Base Medium (Agilent, 30119005) supplemented with 1 mM pyruvate (Sigma, S8636-100 mL), 2 mM glutamine (Sigma, G8540-25g) and 5 mM glucose (Sigma, G8769-100 mL) in a non-CO_2_ incubator at 37 °C for 1 h. During OCR acquisitions, the final concentrations of Oligomycin, FCCP, and Antimycin A were 0.0015 mM, 0.0015 mM, and 0.0005 mM, respectively.

### Determination of malondialdehyde (MDA), superoxide dismutase (SOD), and glutathione peroxidase (GSH-Px) *in vivo*

To quantify the concentrations of MDA and the activities of GSH-Px and SOD, ipsilateral penumbra was homogenized in PBS 24 h after reperfusion and centrifuged at 4 °C, 10,000 rpm for 30 min to obtain supernatants. After that, the kits of MDA (Abcam, ab118970), SOD (Abcam, ab285309) and GSH-Px (MSKBIO, 69-21219) quantification were used to assess concentration or activity according to the manufacturer's instructions.

### Assessment of reactive oxygen species (ROS) *in vitro*

We assessed ROS *in vitro* by using mitoSOX Red (Molecular Probes, Invitrogen, M36008), 2',7'-dichlorodihydrofluorescein diacetate (H2DCF-DA, Solarbio, D6470), and C11-BODIPY (Molecular Probes, Abclonal, RM02821) in accordance with the manufacturer's protocols. Following all interventions, cells were immediately incubated with mitoSOX Red (0.005 mM) for 10 minutes at 37 °C. The fluorescence of mitoSOX Red (Absorption/emission maxima: 510/580 nm) in both HT22 and primary neuronal cells was detected using an Olympus FluoView 1200 confocal microscope (Olympus). H2DCF-DA (Excitation/emission maxima: 488/525 nm) is metabolized by intracellular esterases to a non-fluorescent product, which is oxidized to the fluorescent product CM-DCF, by H_2_O_2_. The cells were washed three times with serum-free cell culture medium to fully remove the DCFH-DA after incubation for 30 min at 37 °C, in a dark incubator, and the ROS levels was analyzed using flow cytometry in primary neuronal cells, and confocal microscope in HT22 cells, respectively. Cells were incubated with 0.002 mM C11-BODIPY (excitation and emission band pass of 460-495 and 510-550, respectively) for 30 min at 37 °C, and the fluorescent density in both HT22 and primary neuronal cells were measured using flow cytometry (FACSCanto II, BD, USA).

### Determination of neurological deficits and infarct volume

The functional consequences of focal cerebral I/R injury were evaluated by neurological severity scoring (NSS) as previously described 23. Briefly, neurological deficits were assessed using a five-point neurological deficit score (0, no deficit; 1, failure to extend the left paw; 2, circling to the left; 3, falling to the left; and 4, unable to walk spontaneously) in a blinded fashion. For infarct volume calculation, mice were sacrificed after the last neurologic deficit score evaluation, and brains were harvested and immediately sliced into 2-mm-thick coronal sections, followed by incubation in 2% 2,3,5-triphenyltetrazolium chloride (TTC, Sigma, T8877-25G) at 37 °C for 30 min. We used the following formula to calculate the percentage of infarct volume: Infarct volume (%) = [contralateral volume (mm^3^) - ipsilateral non-infarct volume (mm^3^)]/[2×contralateral volume (mm^3^)]×100% 31.

### Corner turn test

The corner turn test was performed as our previous study described 23. Briefly, two vertical boards were attached to each other forming an angle of 30°. The mouse was put into the apparatus and tested for the side chosen to leave the corner once it made contact to the boards with its whiskers. Amounts of trials performed per day was 10. Whereas healthy animals leave the corner without side preference, mice suffering from stroke preferentially leave the corner towards the non-impaired body side. We used the following formula: the laterality index = (number of left turns-number of right turns)/10 to assess the laterality index.

### Statistical analysis

We used student's *t*-test to compare two groups. Heat maps were pictured using Gene Cluster 3.0 and Gene Tree View software. Survival analysis was calculated using Kaplan-Meier survival curves and log-rank test. Statistical analyses were performed using GraphPad Prism 8. P < 0.05 was considered statistically significant. All data are presented as the mean ± standard deviation (S.D.) ns, nonsignificant, *P < 0.05, **P < 0.01, or ***P < 0.001.

## Results

### PTRF expression is distinctly elevated in the ischemic penumbra after cerebral I/R injury

**Figure 1:**
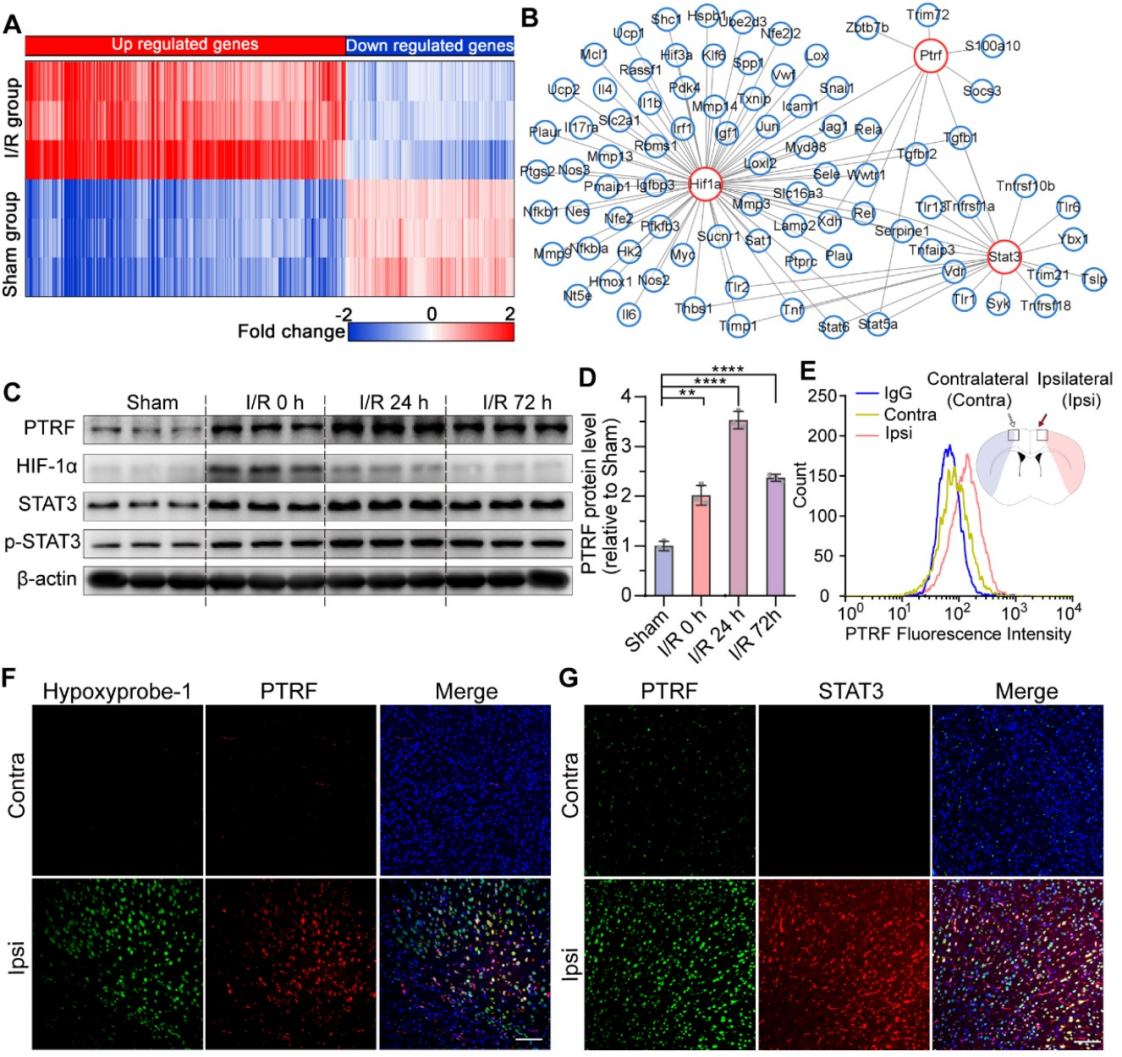
** PTRF is highly expressed in ischemic penumbral regions after cerebral I/R injury. (A)** The heatmap showed the up- and downregulated genes in the sham and I/R groups. **(B)** The network revealed multiple modules of co-expressed genes in I/R groups. **(C)** Western blot of PTRF, HIF-1α, STAT3, and p-STAT3 in the ipsilateral cortical tissue lysates from the mice at 0, 24, and 72 h post-cerebral I/R injury (n = 3). **(D)** Quantification of PTRF normalized to β-actin in the ipsilateral cortical tissue lysates from the mice at 0, 24, and 72 h post-cerebral I/R injury (n = 3). **(E)** Brain cells were collected from the ipsilateral and contralateral penumbra of the mice at 24 h post-cerebral I/R injury and processed for detection of PTRF through flow cytometry (n = 3). **(F)** IF was conducted on brain sections from the mice at 24 h post-cerebral I/R injury using anti-PTRF and hypoxyprobe-1. The nuclei were stained with DAPI. Scale bars = 100 µm. **(G)** IF was conducted on brain sections from the mice at 24 h post-cerebral I/R injury using anti-PTRF and anti-STAT3. The nuclei were stained with DAPI. Scale bars = 100 µm.

Previous studies have reported that PTRF plays a key role in cellular senescence, glucose intolerance, lipid metabolism, and mitochondrial bioenergetics 7-10. To investigate its molecular mechanisms underlying cerebral I/R injury, we explored candidate molecular players that could exert pivotal roles in the pathophysiological metabolic events following cerebral I/R injury by organizing the RNA-seq data into biologically coherent networks. This analysis revealed differentially expressed genes between the sham and cerebral I/R groups (Figure 1A). We subsequently put differential genes into the STRING database and drew a genetic interaction network of PTRF (Figure 1B). Moreover, we established an experimental mouse model of transient middle cerebral artery occlusion (MCAO) using the intraluminal filament technique followed by exposure to reperfusion for 0, 24, and 72 h to further evaluate their PTRF expression modulations in the ischemic penumbral cortex by Western blot analysis. We observed that expression of PTRF was immediately increased at 0 h after cerebral I/R injury, followed by a peak at 24 h (Figure 1C-D). However, the expression of HIF-1α, a positive control that is a master regulator for the adaptive cellular response to oxygen concentration and reportedly abundant during organ response to hypoxia 12, was peaked at 0 h post-cerebral I/R injury and gradually decreased during reperfusion (Figure S1A). The expressions of STAT3 and phosphor (p)-STAT3 were increased at 0 h post-cerebral I/R injury and maintained till 72 h in the cerebral ischemic penumbra (Figure S1B-C).

To further confirm these results, we performed flow cytometry and immunofluorescence (IF) analysis in the mice at 24 h post-cerebral I/R injury. Consistent with these results, flow cytometry analysis of the brain cells collected from the ipsilateral penumbra of the mice following cerebral I/R injury further demonstrated a remarkable increase of PTRF-positive cells in the ipsilateral penumbra (Figure 1E). Using the hypoxia marker pimonidazole (hypoxyprobe-1) and an antibody against STAT3, we further demonstrated that PTRF was strongly increased in the hydroxyprobe-1- and STAT3- stained ischemic penumbra of the mice after cerebral I/R injury (Figure 1F-G). These findings suggested that PTRF expression was upregulated in the ischemic penumbra, thereby implying its potential critical roles in the pathophysiological events of cerebral I/R injury.

### Neuronal PTRF is remarkably increased in the cerebral ischemic penumbra after cerebral I/R injury

**Figure 2:**
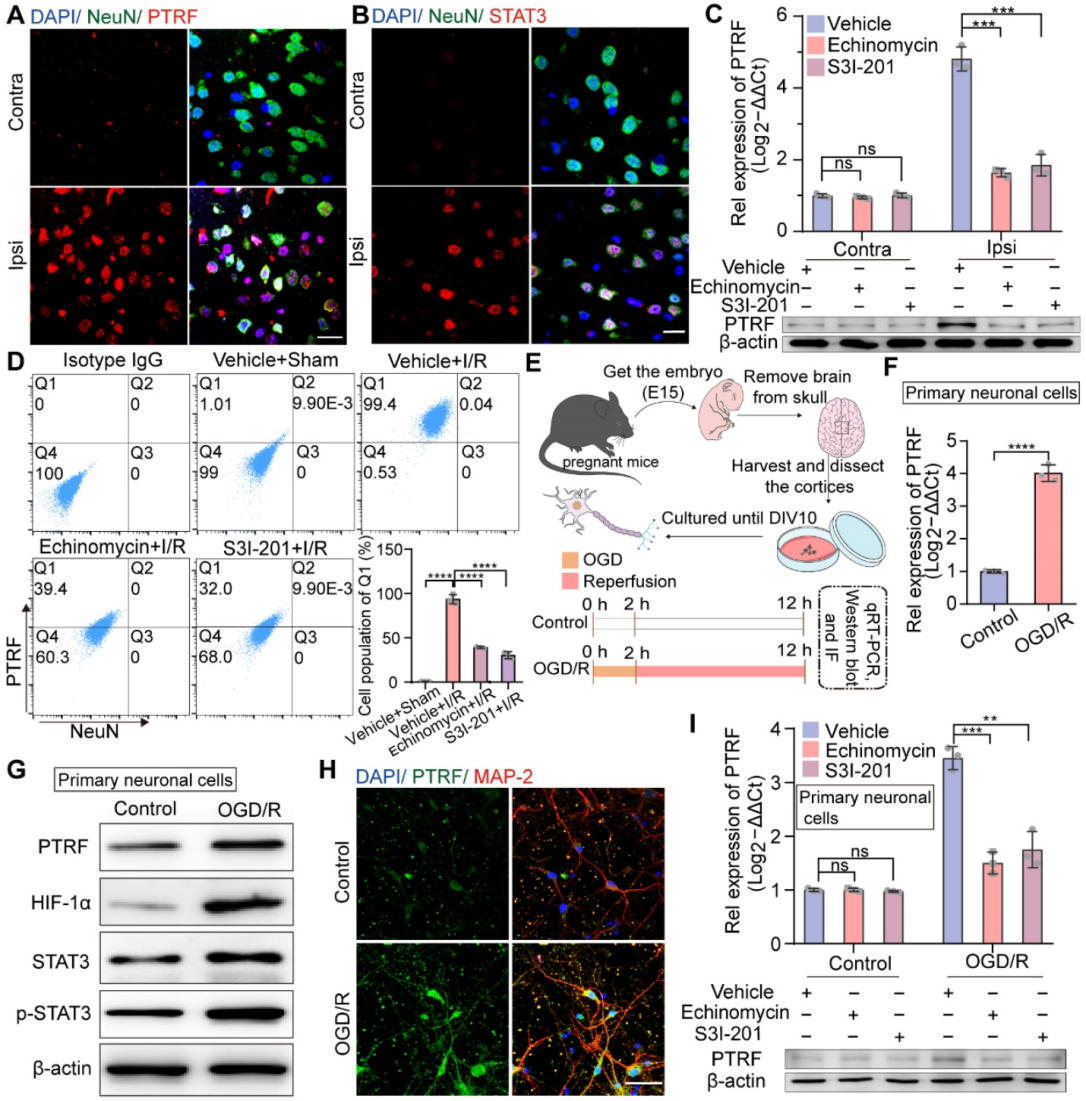
** The expression of neuronal PTRF is evaluated in HIF-1α and STAT3-dependent manners after I/R injury. (A)** IF for the co-localization of NeuN and PTRF in the cortical brain sections from the mice at 24 h post-cerebral I/R injury. Scale bar = 20 µm. **(B)** IF for the co-localization of NeuN and STAT3 in the cortical brain sections from the mice at 24 h post-cerebral I/R injury. Scale bar = 20 µm. **(C)** qRT-PCR and Western blot analyses of the mRNA and protein levels of PTRF in the ipsilateral and contralateral brain of the I/R mice with an intraperitoneal injection of vehicle, echinomycin (10 µg/kg), or S3I-201 (5 mg/kg, n = 3). **(D)** Brain cells were collected from the ipsilateral penumbra of the sham and I/R groups with an intraperitoneal injection of vehicle, echinomycin (10 µg/kg), or S3I-201 (5 mg/kg), processed for simultaneous detection of PTRF and NeuN by using flow cytometry. The histograms summarize the indicated cell populations of PTRF in the neuronal cells (n = 3). **(E)** Upper: Schematic illustration of culture of primary neuronal cells. Lower: The Schematic demonstrating that primary neuronal cells were subjected to OGD for 2 h and reoxygenation for 10 h subsequently. **(F)** qRT-PCR analysis of PTRF levels in primary neuronal cells under OGD/R. **(G)** Representative Western blot analysis of the PTRF, HIF-1α, STAT3, and p-STAT3 in primary neuronal cells under OGD/R. **(H)** IF analysis of PTRF in primary neuronal cells stained with antibodies against neuronal marker MAP2. The nuclei were stained with DAPI. Scale bar = 20 µm. **(I)** qRT-PCR and Western blot analyses of the mRNA and protein levels of PTRF in primary neuronal cells treated with echinomycin (5×10^-6^ mM) or S3I-201 (0.100 mM) under OGD/R (n = 3).

Next, we investigated which cells exhibit PTRF upregulation in the cerebral ischemic penumbra following cerebral I/R injury. Previous studies have indicated that PTRF is highly expressed in a variety of tissues, such as muscle, lung, stomach, and heart, but not in the brain under normal conditions 32. Consistent with previous findings, the IF results demonstrated that PTRF expression was not very significant in neuronal nuclei (NeuN, neuronal cell markers) positive cells in the contralateral cortex but drastically increased in neuronal cells of the ipsilateral penumbra after cerebral I/R injury (Figure 2A). Microglia with high levels of ionized calcium-binding adaptor molecule-1 (Iba-1, an activated microglial marker) and astrocytes with high levels of glial fibrillary acidic protein (GFAP, an activated astrocyte marker) were also found to be significantly populated in the ipsilateral penumbra at 24 h following cerebral I/R injury. Moreover, the IF analysis exhibited that PTRF expressions were not significantly different in activated microglia and astrocytes between ipsilateral and contralateral cerebrum after I/R injury (Figure S2A-B), consistent with our *in vitro* findings at mRNA and protein levels (Figure S2C-E). These results suggested that the expression of PTRF was significantly promoted in neuronal cells of the ischemic penumbra after cerebral I/R injury.

### Neuronal PTRF is upregulated in HIF-1α and STAT3 dependent manners after cerebral I/R injury

Given that not only the cytosolic upregulation of STAT3 level but also its nuclear translocation is promoted in the ischemic core and peri-ischemic regions following cerebral I/R injury 33, consistently we observed significant co-localization of STAT3 and NeuN markers in the ischemic penumbra after cerebral I/R injury by IF analysis (Figure 2B). HIF-1α is considered to be an important regulator of various physiological and pathophysiological conditions, such as cancer and ischemic diseases 11-13. To address whether the participation of HIF-1α and STAT3 was crucial in modulating ischemic stroke-stimulated PTRF expression, echinomycin (inhibitor of HIF-1α) or S3I-201 (inhibitor of STAT3) was injected intraperitoneally (i.p) to mice at the onset of MCAO. Echinomycin or S3I-201 injection resulted in a dose-dependent decrease of the mRNA and protein levels of PTRF in the ipsilateral cortex at 24 h post-cerebral I/R injury (Figure S2F-I and Figure 2C). Echinomycin is a potent small-molecule and cell-permeable inhibitor of HIF-1α DNA-binding activity 34. HIF-1α transcriptionally activity is often determined by the expression levels of HIF-1α target genes such as epo (erythropoietin), vegf (vascular endothelial growth factor), and glut-1 (glucose transporter protein 1) 35, 36. S3I-201 is a specific STAT3 inhibitor that binds to the STAT3-SH2 domain, prevents STAT3 phosphorylation/activation, and inhibits STAT3 dimerization, DNA binding, and target gene activation 37. Moreover, STAT3 plays a critical role in cancer and ischemic stroke by activating genes such as vegf, mmp 9 (matrix metallopeptidase 9), and cyclin D1 (cyclin-dependent kinases) 38, 39. To determine the inhibition effects of echinomycin (10 μg/kg) and S3I-201 (5 mg/kg) *in vivo*, we investigated the mRNA and protein levels of known HIF-1α target genes such as epo, vegf, and glut-1, and that of STAT3 well established target genes such as vegf, mmp 9, and cyclin D1 in the brain tissues from the ipsilateral penumbra of the mice following cerebral I/R injury treated with echinomycin (10 μg/kg) or S3I-201 (5 mg/kg) by qRT-PCR and Western blot analyses (Figure S2J-K).

Furthermore, flow cytometry analysis of the brain cells collected from the ipsilateral cortex after cerebral I/R injury verified that cerebral I/R injury-stimulated PTRF expression was markedly reduced by echinomycin or S3I-201 injection in neuronal cells (Figure 2D). Based on these *in vivo* findings, we next investigated whether neuronal PTRF expression *in vitro* could be stimulated by oxygen-glucose deprivation and reoxygenation (OGD/R), using the primary neuronal cells and HT22 cells. OGD was performed after 10 days of culture of the primary neuronal cells by exposing them to 1% oxygen in a nutrient-free medium for 2 h, followed by re-exposure to ambient air and complete medium for 10 h (Figure 2E), and the expression of PTRF was determined by qRT-PCR and Western blot analyses. We observed that mRNA and protein levels of PTRF were significantly promoted in the primary neuronal cells under OGD/R (Figure 2F-G and Figure S3A-D). IF results further supported that PTRF expression was considerably higher in the primary neuronal cells under OGD/R versus control (Figure 2H). Therefore, these results implied that OGD/R could induce PTRF expression in the primary neuronal cells. In addition, OGD/R-induced PTRF expression was dramatically reduced in the primary neuronal cells treated with echinomycin or S3I-201 (Figure 2I and Figure S3E-H), and the mRNA and protein levels of known HIF-1α target genes such as epo, vegf, and glut-1, and that of STAT3 well established target genes such as vegf, mmp 9, and cyclin D1 were suppressed in the primary neuronal cells treated with echinomycin (5×10^-6^ mM) or S3I-201 (0.100 mM) under OGD/R (Figure S3I-J). Furthermore, echinomycin could decrease the HIF-1α DNA binding activity in the primary neuronal cells under OGD/R (Figure S3K). These results suggested that the overexpression of neuronal PTRF in the primary neuronal cells was in HIF-1α and STAT3 dependent manners under OGD/R.

### HIF-1α and STAT3 bind to the PTRF promoter and regulate its expression in HT22 cells

**Figure 3:**
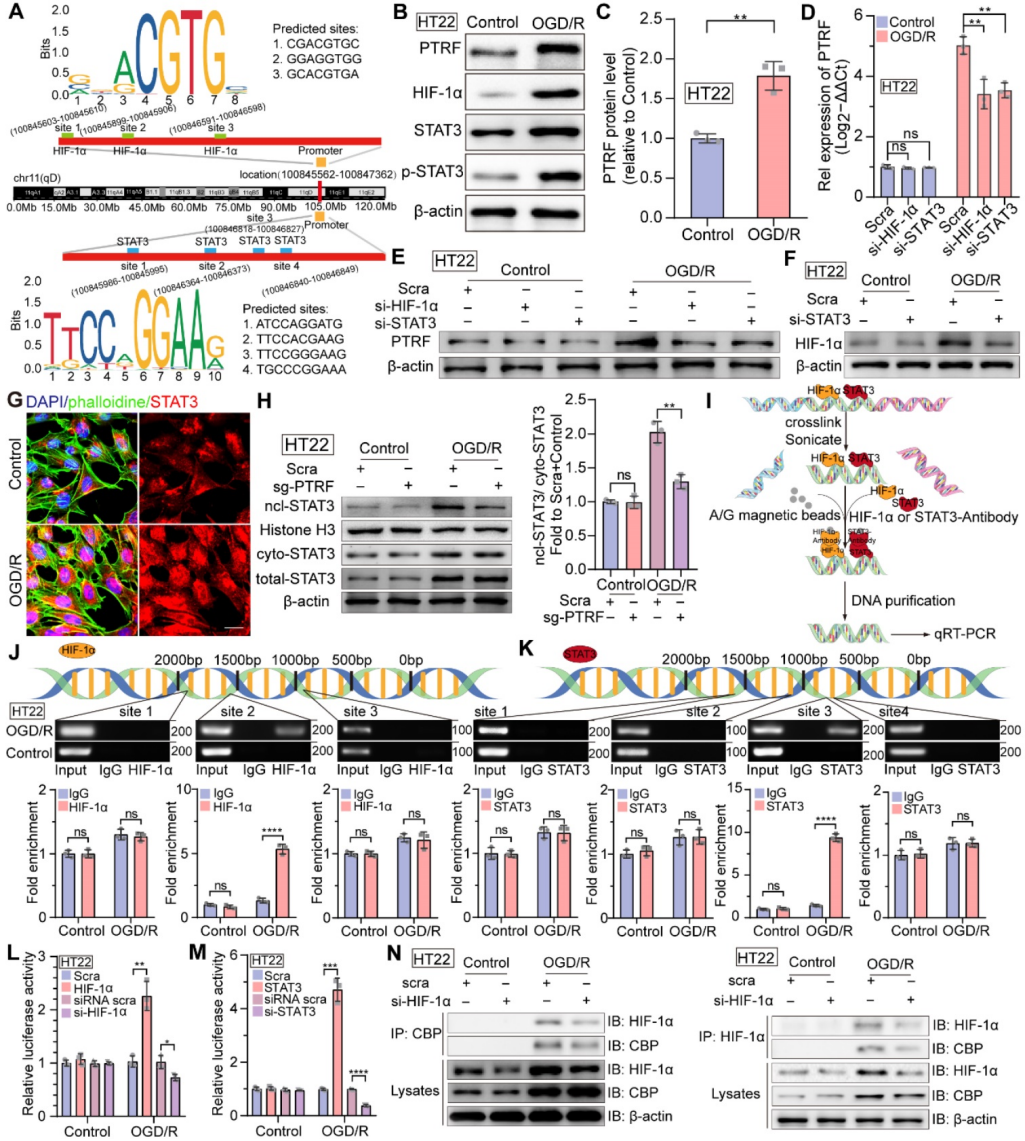
** HIF-1α and STAT3 bind to the *PTRF* gene promoter and regulate its expression in HT22 cells. (A)** Predicted HIF-1α and STAT3-binding sites in the promoter region of *PTRF*. **(B-C)** Western blot analysis of the PTRF, HIF-1α, STAT3, and p-STAT3 in HT22 cells under OGD/R. Quantification of PTRF protein normalized to β-actin in HT22 cells under OGD/R (n = 3). **(D)** qRT-PCR analysis of PTRF in HT22 cells transfected with HIF-1α or STAT3 siRNA under OGD/R. **(E)** Western blot analysis of PTRF in HT22 cells transfected with HIF-1α or STAT3 siRNA under OGD/R. **(F)** Western blot analysis of HIF-1α in HT22 cells transfected with STAT3 siRNA or scramble under OGD/R. **(G)** IF of STAT3 in HT22 cells stained with phalloidine under OGD/R. The nuclei were stained with DAPI. Scale bar = 20 µm. **(H)** Nuclear, cytoplasmic, and total STAT3 proteins were evaluated using Western blot analysis. Histone H3 was used as the nuclear control, and β-actin was used as the cytoplasmic and total control. **(I)** Technical route of ChIP. **(J-K)** ChIP assay of the enrichment of HIF-1α and STAT3 in the *PTRF* promoter region normalized to IgG in HT22 cells under OGD/R. **(L)** Relative luciferase activity following transfection of HIF-1α plasmid or HIF-1α siRNA in HT22 cells under OGD/R (n = 3). **(M)** Relative luciferase activity following transfection of STAT3 plasmid or STAT3 siRNA in HT22 cells under OGD/R (n = 3). **(N)** Cells were lysed from HT22 cells transfected with HIF-1α siRNA or scramble under OGD/R as indicated were processed by IP and Western blot with the indicated antibodies.

A growing body of evidence suggests that both HIF-1α and STAT3 are master transcriptional regulators of numerous genes encoding proteins involved in key aspects of inflammation, mitochondrial metabolism, and metabolic reprogramming in various physiological and pathophysiological conditions 11, 12, 15. Therefore, we performed a bioinformatics analysis of the promoter region of the PTRF and predicted three DNA binding elements (DBEs) for HIF-1α based on the HIF-1α families' binding sites and four DNA binding elements (DBEs) for STAT3 based on the STAT3 families' binding sites in the JASPAR database (Figure 3A). Consistent with above data, PTRF, HIF-1α, STAT3, and p-STAT3 protein levels were increased in the HT22 cells exposed to OGD for 4 h and subsequently re-exposed to ambient air and complete medium for 8 h (Figure 3B-C and Figure S4A-D). OGD/R-induced PTRF expression was decreased in the HT22 cells, following the transfection with anti-sense RNA oligos (siRNA) against HIF-1α or STAT3, and subsequent exposure to OGD/R (Figure S4E-F and Figure 3D-E). Previous studies have revealed that STAT3 regulates the expression of HIF-1α under hypoxic and ischemic conditions, such as tumors and ischemic stroke 16. Consistently, STAT3 KD in the HT22 cells decreased the expression of HIF-1α under OGD/R (Figure S4G and Figure 3F). IF assays showed there was more aggregated STAT3 in the nucleus of HT22 cells under OGD/R compared with that under control (Figure 3G). Williams JJL et al. have uncovered that PTRF decreases interleukin-6 (IL-6) stimulated STAT3 phosphorylation 40, but whether it could regulate the expression of STAT3 and trigger its nucleus translocation remains to be determined. To establish a stable PTRF knockout (KO) HT22 cell line, cells were transduced with lentiviral-Cas9 and subjected to puromycin screening for 2 weeks, followed by transduction with lentiviral-sgRNA against *PTRF* gene (Figure S4H). Western blot results indicated that PTRF could promote nuclear translocation of STAT3 but was unable to regulate the STAT3 expression in HT22 cells under OGD/R conditions (Figure 3H and Figure S4I). Moreover, we used chromatin immunoprecipitation (ChIP)-PCR assay to detect the enrichment of HIF-1α or STAT3 on the *PTRF* gene promoter region (Figure 3I). As shown in Figure 3J-K, both HIF-1α and STAT3 were capable of binding to the promoter region of *PTRF* in the HT22 cells under OGD/R. To further confirm that both HIF-1α and STAT3 could bind to the promoter region of *PTRF*, we transfected HT22 cells with a PTRF luciferase reporter and measured *PTRF* gene promoter activity (Figure 3L-M). Expectedly, *PTRF* promoter activity was prominently enhanced in HT22 cells transfected with HIF-1α or STAT3 plasmids under OGD/R, but it was significantly reduced in HT22 cells transfected with HIF-1α or STAT3 siRNA. These studies suggested that HIF-1α and STAT3 could bind to the PTRF promoter and regulate its expression in HT22 cells under OGD/R, respectively. Furthermore, HIF-1α has been shown to translocate to the nucleus and interact with p300/cyclic adenosine monophosphate (cAMP) response element-binding protein (CREB)-binding protein (CBP) to regulate the transcription of its target genes in cancer 39. Co-IP assay verified that the HIF-1α KD HT22 cells were able to attenuate HIF-1α binding activity with CBP under OGD/R, indicating that HIF-1α could bind with CBP to form a complex in *in vitro* neuronal cells under OGD/R (Figure 3N). Moreover, synergistic induction of HIF-1α and STAT3 conduces to hypoxia-mediated pathophysiological responses 41, and STAT3 is physically in contact with HIF-1α, which is a pivotal factor for activation of HIF-1α target genes in hypoxia as suggested in cancer cells 39, 42. To assess whether HIF-1α could directly interact with STAT3, co-IP experiments were performed in lysates collected from HIF-1α KD HT22 cells under OGD/R (Figure S4J), demonstrating that HIF-1α could not bind to STAT3 in *in vitro* neuronal cells.

### PTRF regulates the activity and stability of PLA2G4A in neuronal cells after I/R injury

**Figure 4:**
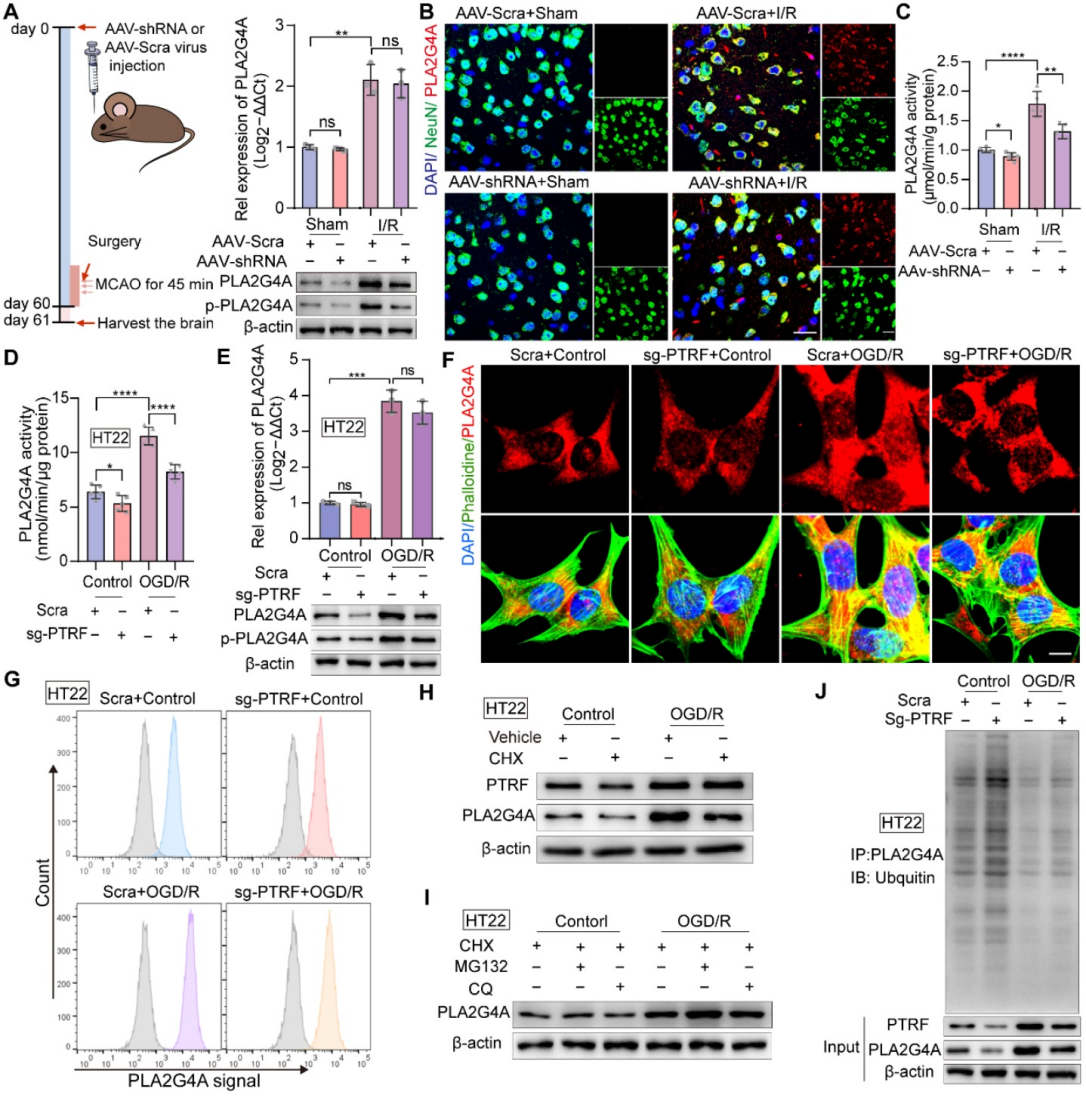
** PTRF regulates the stability and activity of PLA2G4A in neuronal cells after I/R injury. (A)** Left: Schematic for the experimental protocol used. Right: qRT-PCR (upper) and Western blot (lower) analyses of PLA2G4A, and Western blot (lower) analyses of phospho-PLA2G4A in mice transfected with AAV-shRNA or AAV-scramble after I/R injury. **(B)** IF for the co-localization of NeuN and PLA2G4A in the cortical brain sections from mice transfected with AAV-shRNA or AAV-scramble after I/R injury. Scale bar = 50 µm. **(C-D)** PLA2G4A activity in ipsilateral cortical tissues from the mice transfected with AAV-shRNA or AAV-scramble after I/R injury and PTRF KO HT22 cells under OGD/R based on the PLA2G4A assay (n = 5). **(E)** qRT-PCR and Western blot analyses of the mRNA and protein levels of PLA2G4A, and Western blot (lower) analyses of phospho-PLA2G4A in PTRF KO HT22 cells under OGD/R (n = 3). **(F)** IF of PLA2G4A in PTRF KO HT22 cells under OGD/R. Scale bar = 20 µm. **(G)** Flow cytometry analysis of PLA2G4A in PTRF KO HT22 cells under OGD/R. **(H)** Western blot analysis of PTRF and PLA2G4A in HT22 cells treated with cycloheximide (CHX, 0.100 mM). **(I)** Western blot analysis of PLA2G4A in HT22 cells after treatment with CHX (0.100 mM), MG132 (0.010 mM) or chloroquine (CQ, 0.025 mM). **(J)** PTRF KO HT22 cells were lysed to perform co-IP with an antibody against PLA2G4A and analyzed by Western blot with an anti-ubiquitin antibody.

Our previous study has reported that PTRF involves in regulating lipid metabolism reprogramming via stabilizing the PLA2G4A in glioblastoma cells 9. The activity of PLA2G4A is regulated by its phosphorylation via members of the mitogen-activated protein kinase (MAPK) family proteins, making PLA2G4A responsive to extracellular signals 21. PLA2G4A is upregulated and activated in neurodegenerative disorders, brain trauma, spinal cord injury, and ischemic stroke, resulting in neuronal cell death 10, 19-21, 43, 44. Therefore, we hypothesized that neuronal PTRF might be involved in regulating the activity and stability of PLA2G4A following I/R injury. To investigate this *in vivo*, we employed adeno associated virus (AAV) loaded with a short hairpin RNA (shRNA) against PTRF mRNA under control of the neuronal human synapsin (hSyn) core promoter (AAV-hSyn-sh-PTRF) to genetically downregulate the expression of neuronal PTRF in mice through intracerebroventricular (ICV) injection. The AAV enables long-term gene transfer, and the hSyn promoter ensures cell-type-specific restriction of neuronal transgene expression. AAV-mediated neuronal PTRF KD in neuronal cells of the mice following cerebral I/R injury and the primary neuronal cells under OGD/R was confirmed by Western blot (Figure S5A). qRT-PCR analysis showed no significant difference between the mRNA levels of PLA2G4A in the ipsilateral cortical tissues of the I/R mice pre-injected with AAV-hSyn-sh-PTRF and AAV-hSyn-scramble (Figure 4A). However, Western blot analysis showed that the protein and phosphorylation levels of PLA2G4A in neuronal cells were decreased in the ipsilateral cortical tissues of the mice pre-injected with AAV-hSyn-sh-PTRF after cerebral I/R injury (Figure 4A), consistent with IF results of PLA2G4A (Figure 4B). Moreover, the enzymatic activity of PLA2G4A was reduced in the ipsilateral cortical tissues in the mice pre-injected with AAV-hSyn-sh-PTRF after cerebral I/R injury using a cytosolic phospholipase A2 Assay Kit (Figure 4C). Importantly, our *in vitro* results were highly consistent with the* in vivo* findings (Figure 4D-G and Figure S5B-D). In summary, neuronal PLA2G4A mRNA expression occurred in a PTRF-independent manner following I/R injury; however, PTRF regulated the increased protein expression, phosphorylation levels, and enzymatic activity of PLA2G4A, suggesting the possibility of posttranscriptional regulatory interaction between PTRF and PLA2G4A in neuronal cells following I/R injury.

To further elucidate this regulatory interaction, we used cycloheximide (CHX) to inhibit protein synthesis in HT22 cells and primary neuronal cells. Western blot showed that PLA2G4A degradation was significantly higher in HT22 cells and primary neuronal cells treated with CHX, compared with that of vehicle-treated cells (Figure 4H and Figure S5E). The abundance of any protein is determined by the equilibriums between the synthesis and degradation rates of that target protein 45. Regulated intracellular protein degradation has emerged as an efficient means to precisely control the abundance of individual protein within cells and is mainly mediated by the ubiquitin-proteasome system (UPS) and the autophagy-lysosome pathway (ALP) 45. To further investigate the underlying mechanism associated with PTRF and PLA2G4A stability in neuronal cells under OGD/R, MG132 (a proteasome inhibitor), and chloroquine (CQ, a lysosome inhibitor) treatments were performed in both HT22 and primary neuronal cells (Figure 4I and Figure S5F). Consistent with previous results, OGD/R-induced PTRF expression triggered an increase in PLA2G4A protein level when treated with MG132 but not CQ, suggesting that degradation of PLA2G4A was precisely through the proteasome-mediated degradation pathway. Furthermore, co-IP experiments using whole-cell lysates collected from PTRF KO HT22 and PTRF KD primary neuronal cells under OGD/R revealed significantly increased ubiquitination level of PLA2G4A protein (Figure 4J and Figure S5G), indicating that I/R injury-induced neuronal PTRF facilitated the PLA2G4A stability via decreasing its proteasome-mediated degradation by a yet unknown mechanism.

### PTRF regulates lipid metabolism reprogramming and mitochondrial respiration through PLA2G4A in neuronal cells

**Figure 5:**
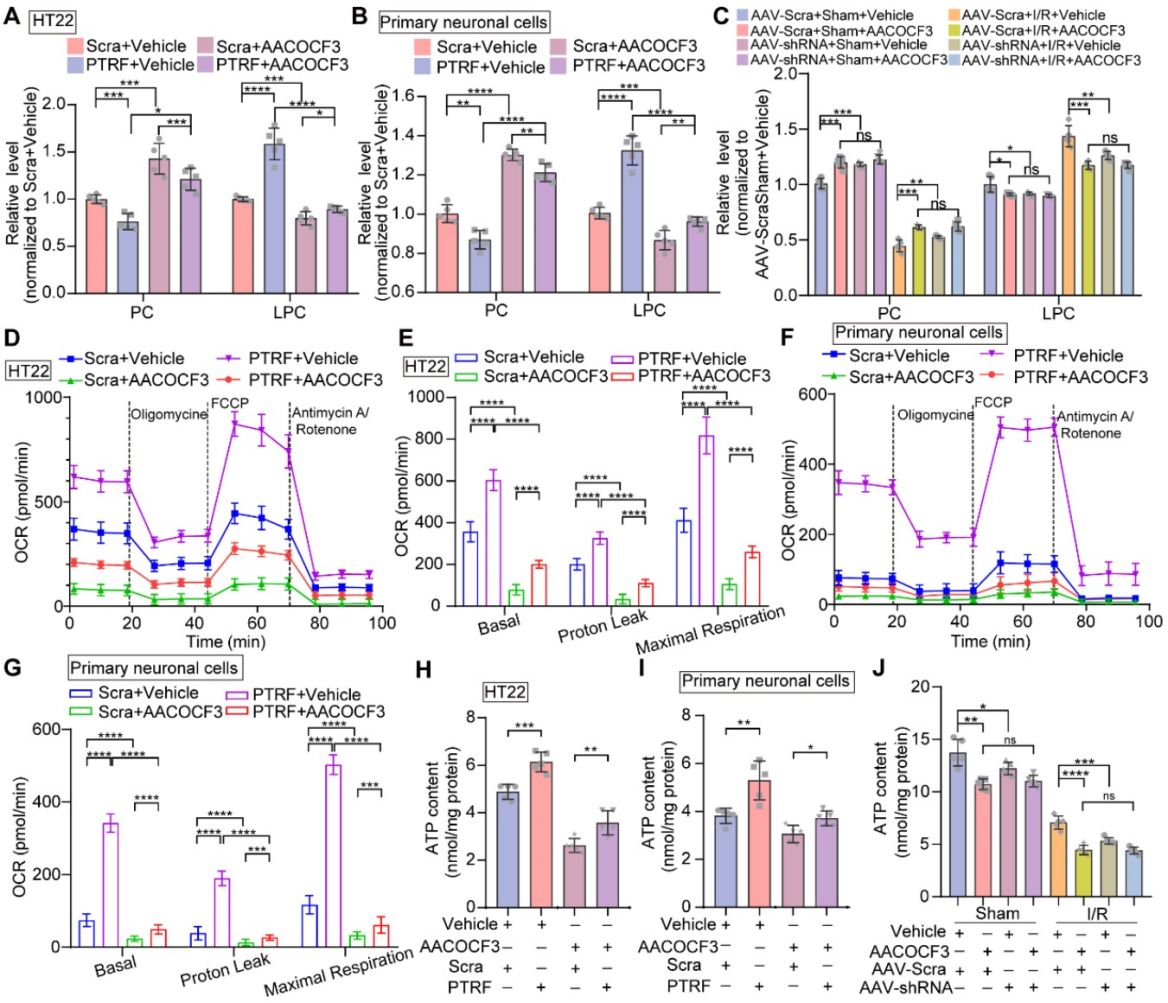
** Overexpression of neuronal PTRF reprograms phospholipid metabolism and regulates mitochondrial respiration via PLA2G4A. (A-B)** The relative level of PC and LPC were determined by using ELISA kits in HT22 and primary neuronal cells after overexpression of PTRF and/or treated with AACOCF3 (HT22: 0.050 mM, primary neuronal cells: 0.010 mM, n = 5). **(C)** The relative levels of PC and LPC were determined by using ELISA kits in the ipsilateral penumbra of AAV-shRNA transfected mice co-treated with AACOCF3 (25 mg/kg body weight) after I/R injury. **(D)** OCR measurements of HT22 cells after overexpression of PTRF and/or treated with AACOCF3 (0.050 mM) using a Seahorse Analyzer. n = 3-5 replicates per group. **(E)** OCR measurements of basal respiration, proton leakage, and maximal respiration in HT22 cells. **(F)** Time series for OCR measurements of primary neuronal cells after overexpression of PTRF and/or treated with AACOCF3 (0.010 mM) using a Seahorse Analyzer. n = 3-5 replicates per group. **(G)** OCR measurements of basal respiration, proton leakage, and maximal respiration in primary neuronal cells. **(H-I)** The intracellular ATP concentration was determined by using an ATP determination kit in HT22 and primary neuronal cells after overexpression of PTRF and/or treated with AACOCF3, and the ATP levels were expressed as nmol/mg protein. **(J)** The ATP concentration was determined in the ipsilateral penumbra of AAV-shRNA transfected the I/R mice co-treated with AACOCF3 (25 mg/kg body weight) after I/R injury.

It has been shown that PTRF regulates lipid metabolism reprogramming and mitochondrial functions in various cell types, such as adipocytes, platelets, and glioblastoma cells 8, 9, 32, 46. PLA2G4A, a regulator of lipid remodeling and mitochondrial bioenergetics, hydrolyzes the fatty acyl linkage in the sn-2 position of the PC to release LPC and FFAs 17, 21. HT22 and primary neuronal cells were stably transfected with a lentivirus carrier to overexpress PTRF (Figure S6A-B) and followed by treatment with arachidonyl trifluoromethyl ketone (AACOCF3), an arachidonic acid analog, to selectively inhibit PLA2G4A 21. We harvested the cells and brain tissues and quantitatively measured the levels of PC and LPC using ELISA kits. Overexpression of PTRF in HT22 and primary neuronal cells decreased the levels of PC and increased that of LPC. However, AACOCF3 treatment significantly reversed the levels in both cells and the brain (Figure 5A-C). Moreover, previous studies have suggested that PLA2G4A plays a critical role in mitochondrial respiration and ATP production in platelets 17. Therefore, we hypothesized that the function of PTRF might be mediated via PLA2G4A and whether lentiviral transfection-mediated PTRF overexpression *in vitro* could increase the mitochondrial respiration, which can be reversed by AACOCF3 treatment. We used the Seahorse metabolic analyzer and measured mitochondrial bioenergetics in both HT22 and primary neuronal cells. Cells were incubated in respective complete medium up to 1 h prior to the measurements and subsequently cultured in assay medium with glucose before the experiment. We found that the overexpression of PTRF significantly increased the basal respiration, proton leakage, and maximal respiration in both HT22 and primary neuronal cells, denoting that PTRF overexpression could enhance the mitochondrial functions. In contrast, AACOCF3 treatment reduced their respiration, proton leakage, and maximal respiration in the* in vitro* neuronal cells (Figure 5D-G), indicating that mitochondrial function could be regulated by the PTRF-PLA2G4A pathway. Subsequently, we detected that intracellular ATP concentrations were increased in HT22 and primary neuronal cells after PTRF overexpression while significantly decreased by AACOCF3 treatment (Figure 5H-I). Moreover, intracellular ATP concentrations were decreased in the mice injected with AACOCF3 after cerebral I/R injury, and intraperitoneal injection of AACOCF3 was no significant difference between the mice pre-injected with AAV-hSyn-sh-PTRF and AAV-hSyn-scramble following cerebral I/R injury (Figure 5J). These findings revealed that the PTRF-PLA2G4A pathway could promote lipid metabolism reprogramming and mitochondrial function in neuronal cells following I/R injury.

### Inhibition of neuronal PTRF decreases autophagy, lipid peroxidation, and ferroptosis via PLA2G4A in neuronal cells after I/R injury

**Figure 6:**
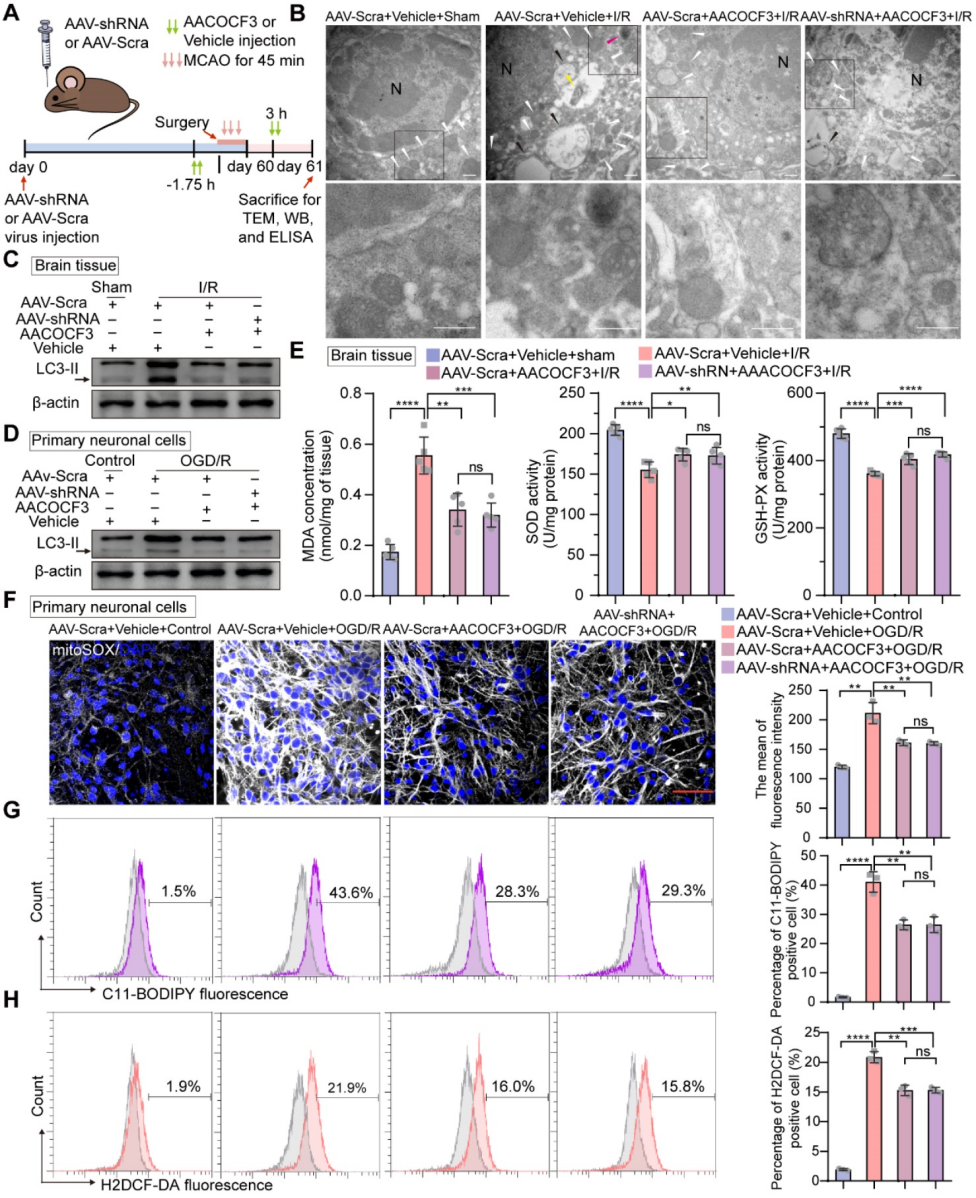
** Inhibition of neuronal PTRF renders cells blunt to autophagy and decreases lipid peroxidation and ferroptosis in neurons via PLA2G4A. (A)** The experimental protocol used. **(B)** TEM of the ipsilateral penumbras from AAV-shRNA transfected mice co-treated with AACOCF3 (25 mg/kg body weight) after I/R injury. White arrowheads, mitochondria; red arrowheads, autophagy; yellow arrowheads, mitophagy; black arrowheads, swelling of cytoplasmic and organelle, as well as plasma membrane rupture. Scale bar = 500 nm. **(C)** Representative Western blot analyses of the LC3-II levels in the ipsilateral penumbras from AAV-shRNA transfected mice co-treated with AACOCF3 (25 mg/kg body weight) after I/R injury. **(D)** Representative Western blot analyses of the LC3-II levels in AAV-shRNA transfected primary neuronal cells co-treated with AACOCF3 (0.010 mM) under OGD/R. **(E)** MDA concentration, SOD, and GSH-Px activities were determined using corresponding kits in the ipsilateral penumbras from AAV-shRNA transfected mice co-treated with AACOCF3 (25 mg/kg body weight) after I/R injury (n = 5). **(F)** Representative images of mitoSOX in AAV-shRNA transfected primary neuronal cells co-treated with AACOCF3 under OGD/R. Neurons were counterstained with DAPI to visualize cell nuclei. Panel right shows the quantification of the mean fluorescence intensity of mitoSOX (n = 3). **(G-H)** Percentage of lipid peroxidation (C11-BODIPY; G) or intracellular ROS levels (H2DCF-DA; H) in AAV-shRNA transfected primary neuronal cells co-treated with AACOCF3 under OGD/R by flow-cytometry analysis (n = 3).

Emerging evidence has revealed that PLA2G4A-mediated lipid metabolism contributes to autophagy and ROS generation after ischemic stroke 10, 21, 22. Moreover, following cerebral I/R injury, sudden nutrient deprivation contributes to energetic stress that, in turn, promotes ATP demand, mitochondrial bioenergetics, and the mitochondrial stress burden, leading to the increase of electron leakage and ROS production 47. Thus, we hypothesized that PTRF could regulate autophagy in neuronal cells after I/R injury via PLA2G4A. Transmission electron microscopy (TEM) revealed a high presence of autophagosomes in the cerebral ischemic penumbra of the mice following cerebral I/R injury, whereas the stroke-triggered accumulation of autophagosomes was no significant difference in the cerebral ischemic penumbra between the AAV-hSyn-sh-Scra and AAV-hSyn-sh-PTRF pre-injected mice co-treated with AACOCF3 after cerebral I/R injury, consistent with the* in vitro* observations (Figure 6A-B and Figure S7A)*.* Western blot was performed to further detect the expression of autophagy-associated protein in the ipsilateral cerebral cortex at 24 h post-I/R injury, which revealed that there was no significant difference in the expression of MAP1LC3-II/LC3-II (autophagic markers) 48 in the ipsilateral cerebral cortex between the AAV-hSyn-sh-Scra and AAV-hSyn-sh-PTRF pre-injected mice co-treated with AACOCF3 after cerebral I/R injury (Figure 6C). Consistently, the expression of LC3-II protein was increased *in vitro* under OGD/R, while there had no significant difference between non-PTRF and PTRF inhibition in the *in vitro* neuronal cells both co-treated with AACOCF3 (Figure 6D and Figure S7B). Together, these results suggested that there may be an abnormal accumulation of autophagosomes in neuronal cells after cerebral I/R injury mediated by PTRF/PLA2G4A axis.

Polyunsaturated fatty acids (PUFAs) are highly susceptible to the noxious lipid peroxidation chain reaction in the brain neuronal cells, enriched in oxidizable unsaturated fatty acids 4. Ferroptosis, characterized by the iron-dependent accumulation of lipid hydroperoxides to lethal levels, is a form of regulated cell death 49. PLA2G4A is closely associated with the pathophysiology of neurological diseases via participating in oxidative stresses, such as autophagy, lipid peroxidation, and ferroptosis 4, 19, 20. As indicated in Figure 6E, compared to the sham group, mice subjected to cerebral I/R injury showed an obvious increase in malondialdehyde (MDA) level, a frequently used membrane lipid peroxidation hallmark 5. Moreover, MDA level was significantly lower in the ipsilateral cerebral cortex of the mice injected with AACOCF3 than vehicle after cerebral I/R injury, while there was no significant difference between AAV-hSyn-sh-PTRF- and AAV-hSyn-scramble-pre-transfected mice co-treated with AACOCF3. In addition, the activities of glutathione peroxidase (GSH-Px) and superoxide dismutase (SOD), the most important indicators of ferroptosis 50, were significantly higher in mice with injection of AACOCF3 compared with vehicle after cerebral I/R injury. However, there was no significant difference between AAV-hSyn-sh-PTRF- and AAV-hSyn-scramble-pre-transfected mice both co-treated with AACOCF3. To further confirm that PTRF/PLA2G4A-axis regulated lipid peroxidation and ferroptosis *in vitro*, we measured the ROS level by MitoSOX (a probe of mitochondrial ROS), C11-BODIPY (a lipid peroxide indicator), and H2DCF-DA (a probe of intracellular reactive oxygen species) labeling to monitor the ferroptosis in HT22 and primary neuronal cells under OGD/R (Figure 6F-H and Figure S7C-E). Interestingly, OGD/R induced a dramatic steep increase in ROS levels in both HT22 and primary neuronal cells, which were subsequently attenuated by AACOCF3 treatment. However, there were no significant differences in ROS levels as measured by MitoSOX, C11-BODIPY, and H2DCF-DA between non-PTRF and PTRF inhibited cells co-treated with AACOCF3. Collectively, these results indicated that inhibition of PTRF could render neuronal cells blunt to autophagy and decrease lipid peroxidation and ferroptosis via PLA2G4A *in vivo* and *in vitro* after cerebral I/R injury.

### Inhibition of neuronal PTRF prevents brain damage after I/R injury

**Figure 7:**
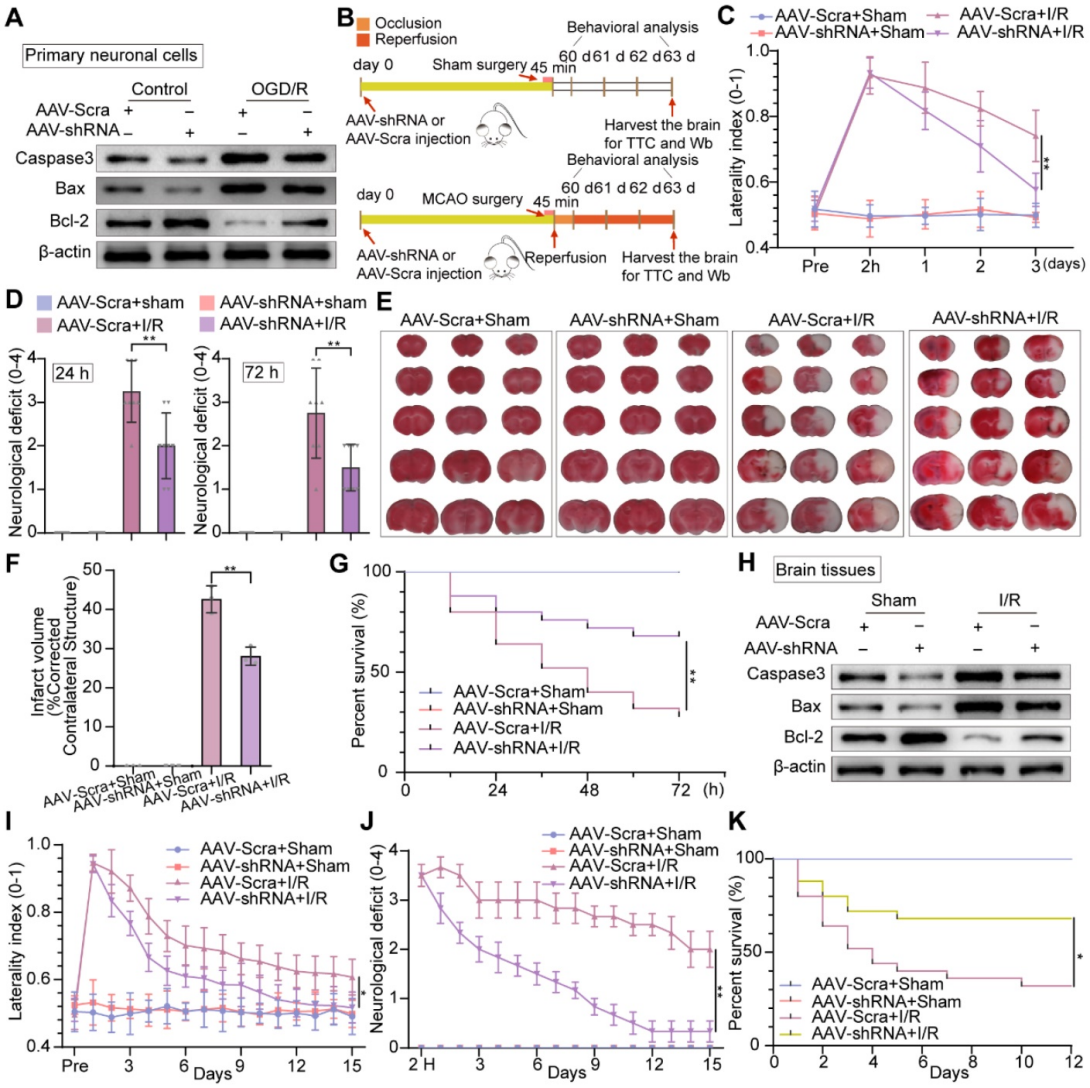
** Knockdown of neuronal PTRF protects brain against cerebral I/R injury. (A)** Western blot was used to analyze the expression levels of Caspase3, Bax, and Bcl-2 in AAV-shRNA transfected primary neuronal cells under OGD/R. **(B)** The experimental protocol used in mice transfected with AAV-shRNA or AAV-Scra after I/R injury. **(C)** The corner turn test was analyzed using the laterality index (n = 6). **(D)** Neurological deficit was assessed throughout recovery at 24 h and 72 h post-cerebral I/R injury (n = 8). **(E-F)** TTC stained brain slices showing infarct volume (white), and the infarct volume was measured in the mice transfected with AAV-shRNA or AAV-Scramble after cerebral I/R injury (n = 3). **(G)** Survival rates at 3 d in the mice transfected with AAV-shRNA or AAV-Scramble after cerebral I/R injury (n = 25). **(H)** The expression levels of Caspase3, Bax, and Bcl-2 were measured by Western blot analysis in the ipsilateral penumbras from AAV-shRNA or AAV-Scramble transfected mice after I/R injury. **(I)** The corner turn test was analyzed using the laterality index at 15 d post-cerebral I/R injury in mice (number of left turns -number of right turns)/10 (n = 6). **(J)** Daily neurological deficit score was assessed over the 15 recovery days (n = 6). **(K)** Mouse survival rates at 15 d post-cerebral I/R injury in mice (n = 25).

To address whether PTRF deteriorated the neuronal damage under OGD/R *in vitro*, we performed Western blot to analyze the expressions of the activation of cysteine proteases (Caspase-3), the B-cell lymphoma 2-associated X protein (Bax), and B cell lymphoma-2 (Bcl-2) in primary neuronal cells and HT22 cells under OGD/R (Figure 7A and Figure S8A). The expressions of Caspase-3 and Bax were increased, while Bcl-2 expression was reduced* in vitro* under OGD/R, and these results were reversed by PTRF inhibition. To further verify if PTRF exacerbated neuronal insults after ischemic stroke *in vivo* (Figure 7B), we use of the corner turn test to quantify neurological severity in the mice after cerebral I/R injury, which showed that the lower percentage of left turn was calculated in AAV-hSyn-sh-PTRF-transfected mice (Figure 7C). Moreover, there was a sustained reduction of neurological deficits after 3 days in AAV-hSyn-sh-PTRF-transfected mice after cerebral I/R injury (Figure 7D). Staining with 2% triphenyltetrazolium chloride (TTC) showed that cerebral I/R injury was attenuated in AAV-hSyn-sh-PTRF-transfected mice (Figure 7E). The infarction and mortality were significantly reduced in this mice group, compared with that of the AAV-hSyn-scramble transfected mice on day 3 post-I/R injury (Figure 7F-G). Furthermore, Western blot analyses of Caspase-3, Bax, and Bcl-2 proteins in the ipsilateral cerebral cortex of the mice following I/R injury were consistent with that of *in vitro* results (Figure 7H). Subsequently, we demonstrated that AAV-hSyn-sh-PTRF-transfected mice had better performance on the corner turn tests, lower acute neurological deficits, and mortality compared to mice transfected with AAV-hSyn-scramble. In addition, AAV-hSyn-sh-PTRF-transfected mice also showed a greater improvement in recovery of initial deficit over the 15-day period (Figure 7I-K). Our findings thus illustrated that inhibition of neuronal PTRF could effectively reduce brain injury in both *in vitro* and *in vivo* experimental I/R models.

### Inhibition of neuronal PTRF/PLA2G4A-axis facilitates neuroprotection after cerebral I/R injury

**Figure 8:**
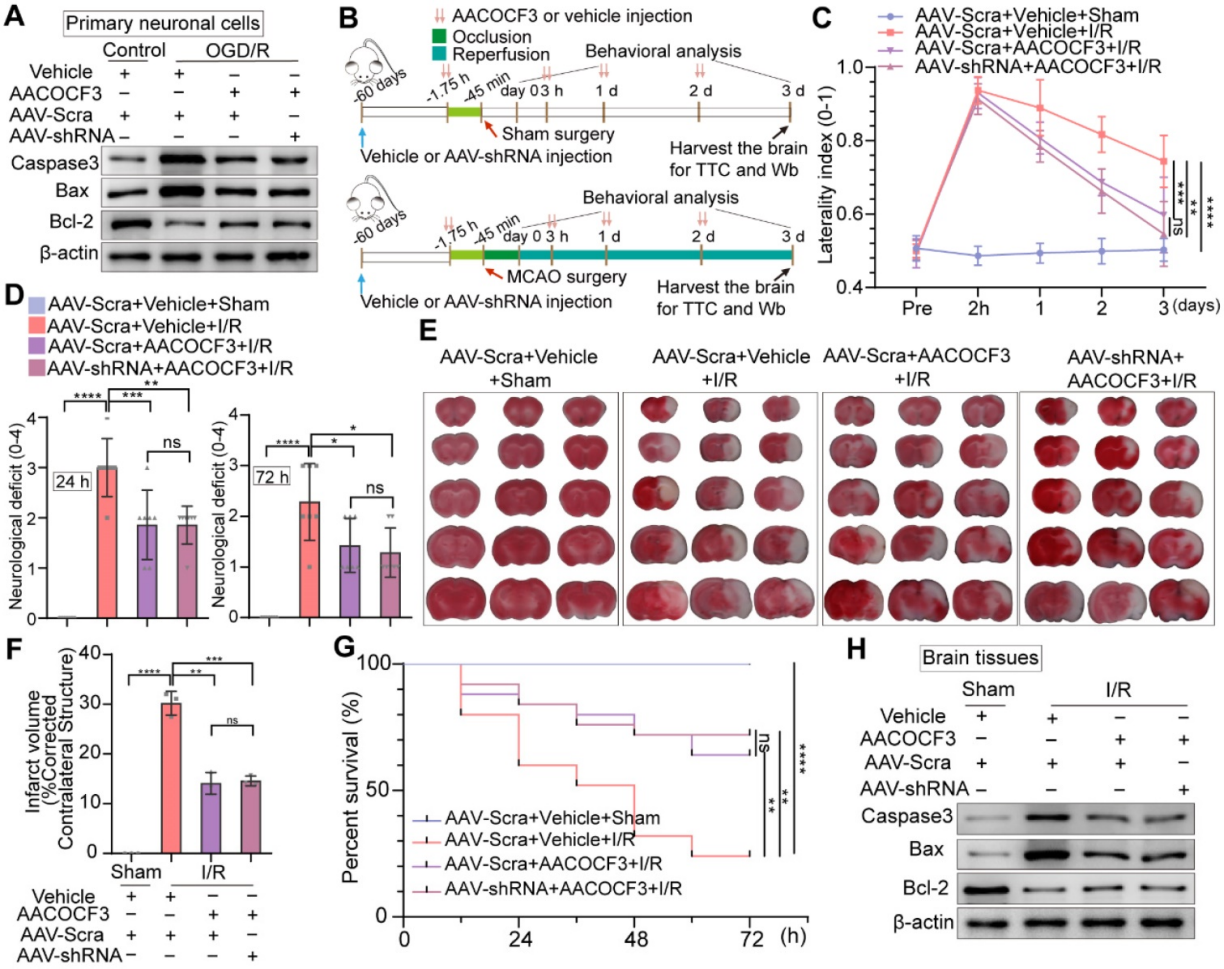
** Knockdown of neuronal PTRF does not enhance PLA2G4A inhibition induced neuroprotection after cerebral I/R injury. (A)** Western blot analyses of the expression levels of the Caspase3, Bax, and Bcl-2 in AAV-shRNA transfected primary neuronal cells co-treated with AACOCF3 (0.010 mM) under OGD/R. **(B)** The experimental protocol used in AAV-shRNA transfected mice co-treated with AACOCF3 (25 mg/kg body weight) after I/R injury. **(C)** The corner turn test was analyzed using the laterality index in AAV-shRNA transfected mice co-treated with AACOCF3 (25 mg/kg body weight) after I/R injury (n = 6). **(D)** Neurological deficit was assessed throughout recovery in the mice at 24 h and 72 h post-cerebral I/R injury (n = 7). **(E-F)** TTC stained brain slices showing infarct volume (white), and the infarct volume was measured in AAV-shRNA transfected mice co-treated with AACOCF3 (25 mg/kg body weight) after I/R injury (n = 3). **(G)** Mouse survival rates at 3 d post-cerebral I/R injury in the mice (n = 25). **(H)** The expression levels of Caspase3, Bax, and Bcl-2 in the ipsilateral penumbras of the mice after cerebral I/R injury.

To further determine if inhibition of PTRF could promote neuroprotective effects via regulating PLA2G4A, we detected the expressions of apoptotic and anti-apoptotic proteins in primary neuronal cells and HT22 cells treated with AACOCF3 under OGD/R. AACOCF3 treatment exhibited reduced expressions of Caspase-3 and Bax and increased Bcl-2 level in both primary neuronal cells and HT22 cells under OGD/R, and no significant difference was observed when AAV-hSyn-sh-PTRF- and CRISPR/Cas9-transfection was introduced to *in vitro* neuronal cells co-treated with AACOCF3 (Figure 8A and Figure S8B). To further assess whether PTRF expression could exacerbate the neuronal insults through PLA2G4A after cerebral I/R injury *in vivo* (Figure 8B), we investigated the neurological severity using the corner turn test, and neurological deficit assessments for three days. A decrease of the percentage of a left turn and the neurological scores were observed in AACOCF3-treated mice, and no significant difference was observed between AAV-hSyn-sh-PTRF- and AAV-hSyn-scramble-pre-transfected mice with AACOCF3 co-treatment (Figure 8C-D). Furthermore, the infarct volume and mortality were decreased in the AACOCF3-treated mice group at day 3 post-cerebral I/R injury, and no difference was observed between AAV-hSyn-sh-PTRF- and AAV-hSyn-scramble-pre-transfected mice co-treated with AACOCF3 (Figure 8E-G). Western blot analyses of Caspase-3, Bax, and Bcl-2 proteins were consistent with that of *in vitro* findings (Figure 8H). Therefore, these results indicated that inhibition of PTRF/PLA2G4A-axis could protect the brain from cerebral I/R injury.

## Discussion

Clinical and preclinical findings indicate that bioenergetic disturbances and redox imbalance are implicated in the pathogenesis of ischemic stroke, and patients with such pathological hallmarks exhibit poorer outcomes 5, 50. In this study, we explored the mechanisms among the bioenergetic disturbance, redox dyshomeostasis, and brain damage following cerebral I/R injury.

Accumulating evidence reveals that PTRF is an abundant component of caveolae and a critical factor in the function of caveola 7. Recent studies have further shown that PTRF regulates lipid metabolism reprogramming and mitochondrial bioenergetics in CGL and glioblastoma 8-10, 17, 32, 43, 51. Our experiments revealed that PTRF expression was elevated following cerebral I/R injury, predominantly in neuronal cells of penumbral regions, which mainly generates ROS 5, and there was a correlation between HIF-1α/STAT3 expressions and PTRF.

HIF-1α is recognized as a critical physiological regulator of the cellular transcriptional response to hypoxia 12, 13. STAT3, a latent transcription factor, regulates the activation of genes that are involved in mitochondrial functions and cellular metabolisms. It also regulates the HIF-1α mRNA level during the ischemic stroke 14, 15. In addition, HIF-1α and STAT3 have been suggested to play critical roles in cerebral I/R injury and proposed as a therapeutic target for ischemic stroke 14, 15. Given our observations that expression of neuronal PTRF was upregulated following cerebral I/R injury, we further confirmed that PTRF overexpression was mediated in HIF-1α and STAT3-dependent manners in the mice following cerebral I/R injury and primary neuronal cells under OGD/R by using echinomycin and S3I-201. Previous studies have reported that echinomycin targets AML (Acute myelocytic leukemia) blasts which show no detectable HIF activity under nomoxia at steady state due to an off-target effect 52. We used HIF-1α Transcription Factor Assay Kit to investigate the HIF-1α DNA binding activity in the primary neuronal cells treated with echinomycin (5×10^-6^ mM), and found echinomycin could reduce the HIF-1α DNA binding activity in the primary neuronal cells under OGD/R. S3I-201 is highly selective for the SH2 domain of STAT3, which disrupts STAT3-STAT3 interaction through SH2 domain binding 37. However, few reports about off-target effects of S3I-201 have been reported. These correlations between HIF-1α/STAT3 expressions and PTRF were further confirmed in HT22 cells transfected with siRNA against HIF-1α or STAT3. PTRF is a critical component of caveolae and plays a specialized role in lipid raft microdomains within the phospholipid membrane 7. Previous studies have demonstrated that cytokine receptors of STAT3 are localized in lipid raft microdomains, and PTRF deletion triggers an increase in IL-6-stimulated Tyr705 STAT3 phosphorylation 40. Our experiments strongly supported the fact that PTRF KO in HT22 cells might critically decrease the nuclear translocation of STAT3 under OGD/R, but did not change the mRNA and protein levels of STAT3. Moreover, ChIP-PCR and luciferase activity assays showed that HIF-1α and STAT3 could bind to the *PTRF* gene promoter and subsequently regulated its transcriptional activity in HT22 cells under OGD/R. Furthermore, HIF-1α could recruit the transcriptional activators CBP, without interacting with STAT3, suggesting that HIF-1α could interact with CBP to regulate the expression of PTRF and both the factors of HIF-1α and STAT3 could enhance the transcription of PTRF independently. These results demonstrated that PTRF was a downstream mediator of HIF-1α and STAT3 and had positive feedback for the nuclear translocation of STAT3 in neuronal cells after I/R injury.

Cell type-specific expression and activation of PLA2G4A have been confirmed in the cortex following brain trauma 21. The initial neuron-specific activation of PLA2G4A is involved in neuronal cell death, whereas its later activation (day 3) is predominantly associated with inflammatory responses in microglial cells 21. It has been reported that PTRF regulates lipid metabolism remodeling by stabilizing PLA2G4A in glioblastoma 9. Of note, we found that neuronal PTRF had no effect on the mRNA level of PLA2G4A, but regulated the protein and phosphorylation levels of PLA2G4A after I/R injury, suggesting the possibility of posttranscriptional regulatory interaction between PTRF and PLA2G4A in neuronal cells following I/R injury. Moreover, we revealed that the posttranscriptional regulatory interaction between PTRF and PLA2G4A was that PTRF enhanced the activity and stability of PLA2G4A by decreasing the proteasome-mediated PLA2G4A degradation in neurons following cerebral I/R injury. During ischemic stroke, the brain challenges acute energy failure triggering a complex series of metabolic events that ultimately contribute to neuronal cell death 20. One such critical metabolic event is PLA2G4A activation promoting lipid metabolism reprogramming that leads to the hydrolysis of membrane PC to release LPC and FFAs 4, 18, which in turn causes an imbalance between PC and LPC levels and generates ROS to further aggravate the brain damage after the I/R injury 10. PLA2G4A is reported as the key regulator of lipid remodeling and mitochondrial bioenergetics 17, 21. We found that PTRF overexpression promoted lipid metabolism remodeling and altered mitochondrial bioenergetics via PLA2G4A in neuronal cells, which was reversed by AACOCF3 treatment.

Neuronal cells are particularly vulnerable to oxidative damage due to their high consumption of oxygen, rich content of iron and PUFAs, and relatively low capacity of endogenous antioxidant 4. Nutrient deprivation leads to energetic stress that, in turn, causes the increase of ATP demand, leading to mitochondrial overburden, thereby increasing electron leakage and ROS production after cerebral I/R injury 47. PLA2G4A activation could facilitate oxidative damages to the membrane, which results in membrane disruption and cell death via autophagy, lipid peroxidation and ferroptosis following cerebral I/R injury 19-22, 53, 54. In this study, we found that ischemia-induced neuronal PTRF expression could lead to autophagy, lipid peroxidation, and ferroptosis via PLA2G4A after cerebral I/R injury. Furthermore, PTRF KD improved the functional neurological deficits in the mice transfected with AAV-hSyn-sh-PTRF after cerebral I/R injury, while no significant difference was observed between AAV-hSyn-sh-PTRF- and AAV-hSyn-scramble-pre-transfected mice with AACOCF3 co-treatment. These results indicated that inhibition of neuronal PTRF/ PLA2G4A-axis could protect the brain against cerebral I/R injury.

In general, we presented a molecular player, PTRF, that linked disturbances of mitochondrial bioenergetics and redox homeostasis during cerebral I/R injury by stabilizing PLA2G4A. We revealed that neuronal PTRF was increased in HIF-1α and STAT3-dependent manners under *in vitro* and* in vivo* I/R injury, and inhibition of PTRF/ PLA2G4A-axis reduced the infarct volume and neurological deficits in cerebral I/R models. Therefore, the neuronal STAT3/HIF-1α/PTRF-axis that aggravate cerebral I/R injury by PLA2G4A, might be a potential therapeutic target for cerebral I/R injury.

## Supplementary Material

Supplementary figures and tables.

## Abbreviations

PTRF: polymerase I and transcript release factor
I/R: ischemia-reperfusion
AAV: adeno-associated virus
PLA2G4A: phospholipase A2, group IVA [cytosolic, calcium-dependent]
ROS: reactive oxygen species
HIF-1α: hypoxia-inducible transcription factor 1α
STAT3: signal transducer and activator of transcription 3
PC: phosphatidylcholine
LPC: lysophosphatidylcholine
FFA: free fatty acids
KD: knockdown
MCAO: middle cerebral artery occlusion
OGD/R: oxygen and glucose deprivation/reoxygenation
qRT-PCR: quantitative real-time polymerase chain reaction
IF: immunofluorescence
CHX: cycloheximide
CQ: chloroquine
AACOCF3: arachidonyl trifluoromethyl ketone
ChIP: chromatin immunoprecipitation
co-IP: co-immunoprecipitation
MDA: malondialdehyde
SOD: superoxide dismutase
GSH-Px: glutathione peroxidase
KO: knockout
PUFAS: polyunsaturated fatty acids
EPO: erythropoietin
VEGF: vascular endothelial growth factor
Glut-1: glucose transporter protein 1
MMP 9: matrix metallopeptidase 9
cyclin D1: cyclin-dependent kinases
Caspase-3: cysteine proteases
Bax: the B-cell lymphoma 2-associated X protein
Bcl-2: B cell lymphoma-2
