# Supplementary material for: Neuronal STAT3/HIF-1α/PTRF axis-mediated bioenergetic disturbance exacerbates cerebral ischemia-reperfusion injury via PLA2G4A
Source: Theranostics. 2022 Apr 4;12(7):3196–216. doi: 10.7150/thno.71029 (PMC9065197; doi:10.7150/thno.71029)
Supplement: Supplementary file 1 — Supplementary figures and tables. [file thnov12p3196s1.pdf]

Neuronal STAT3/HIF-1α/PTRF axis-mediated bioenergetic disturbance exacerbates cerebral ischemia-reperfusion injury via PLA2G4A

Weili Jin[1, 2], Jixing Zhao[1, 2], Eryan Yang[1, 2], Yunfei Wang[1, 2], Qixue Wang[1, 2], Ye Wu[1, 2], Fei Tong[1, 2], Yanli Tan[3], Junhu Zhou[1, 2], and Chunsheng Kang[1, 2*]

Affiliations

1. Department of Neurosurgery, Tianjin Medical University General Hospital, Tianjin 300052, China.
2. Tianjin Neurological Institute, Key Laboratory of Post-neurotrauma Neuro-repair and Regeneration in Central Nervous System, Ministry of Education, Tianjin City, Tianjin 300052, China.
3. Department of Pathology, Affiliated Hospital of Hebei University, Baoding 071000, China.

*Correspondence: Chunsheng Kang (kang97061@tmu.edu.cn)

These authors contributed equally: Weili Jin, Jixing Zhao, and Eryan Yang

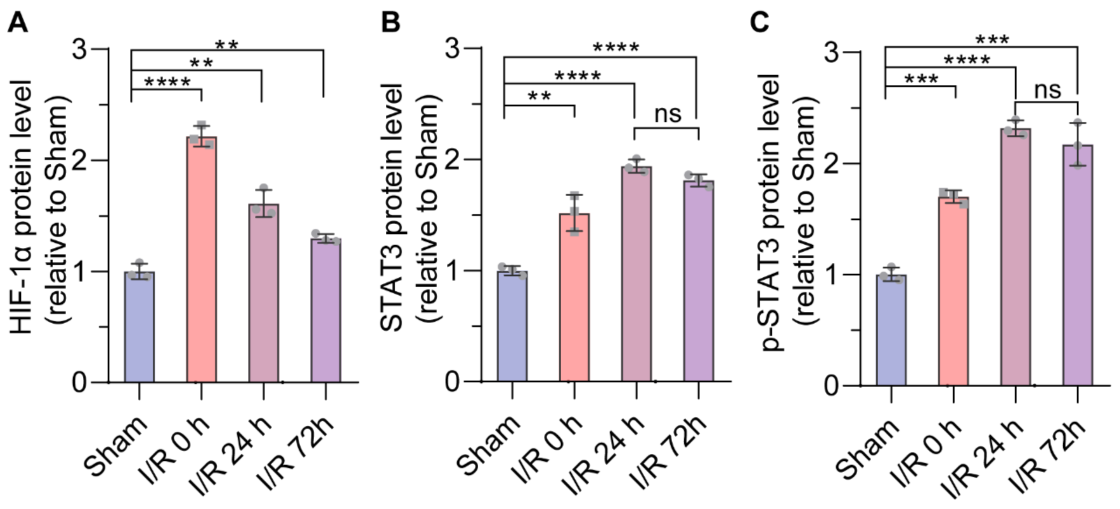

Figure S1. Quantification of HIF-1α, STAT3, and p-STAT3 proteins in the ipsilateral cerebral cortex of the mice after cerebral I/R injury.

A-C. Quantification of HIF-1α, STAT3, and p-STAT3 proteins normalized to β-actin in the ipsilateral cerebral lysates from the mice at 0, 24, and 72 h post-I/R injury (n = 3).

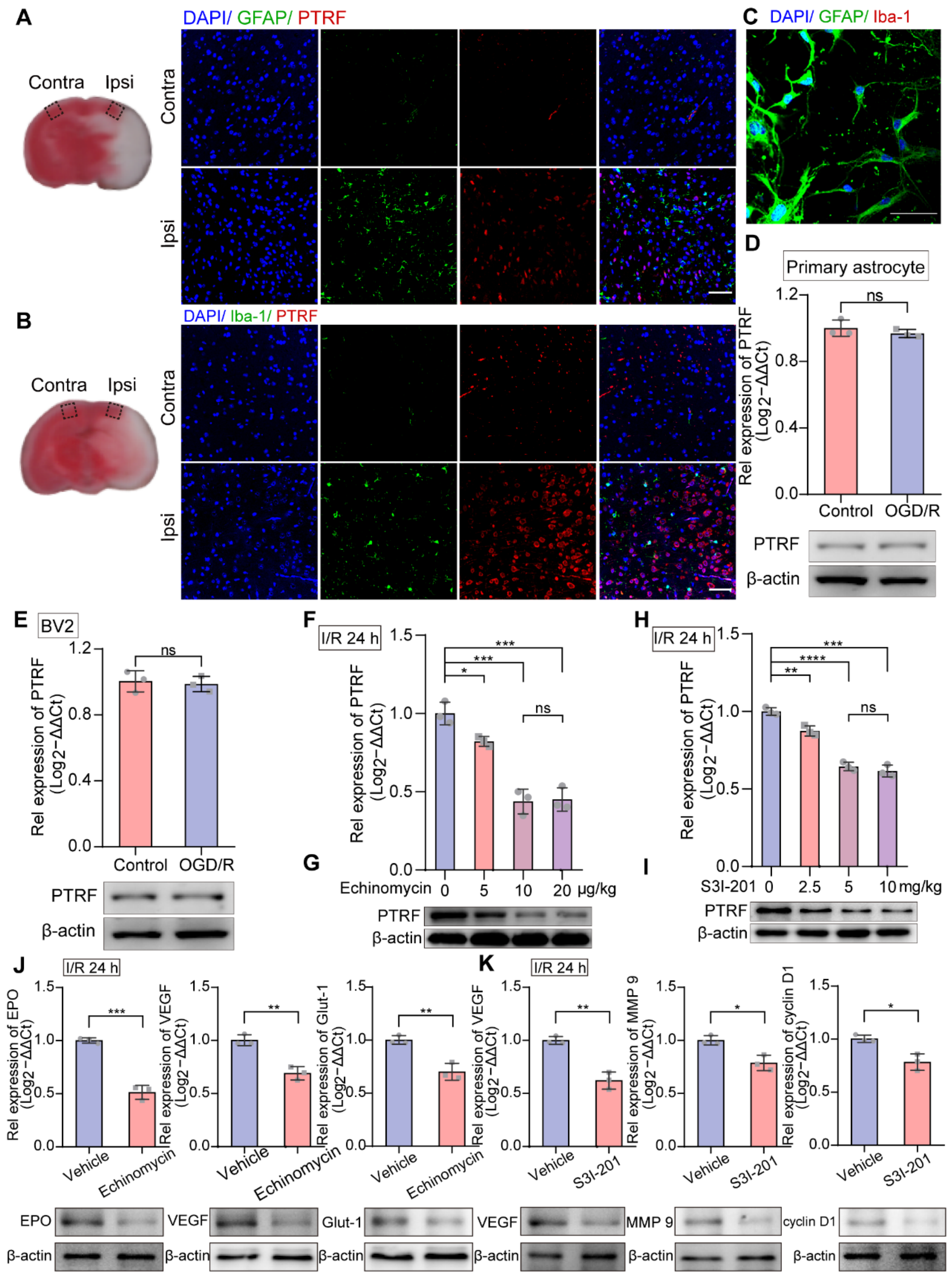

Figure S2. Expression of PTRF in the *in vitro* and *in vivo* astrocytes and microglia after I/R injury and it is evaluated in HIF-1α and STAT3 dependent manners in the ipsilateral cerebral cortex.

A. IF was conducted on coronal sections from the mice at 24 h post-cerebral I/R injury

using antibodies against GFAP and PTRF. Scale bar = 50 μm.

B. IF was performed on coronal sections from the mice at 24 h post-cerebral I/R injury using antibodies against Iba-1 and PTRF. Scale bar = 50 μm.

C. Fluorescence imaging of the primary astrocyte. DAPI, and anti-GFAP antibody were used to stain the nuclei, and primary astrocytes. Scale bar = 50 μm.

D. Relative mRNA and protein levels of PTRF, as determined by qRT-PCR and Western blot in primary astrocytes under OGD/R.

E. Relative mRNA and protein levels of PTRF, as determined by qRT-PCR and Western blot in BV2 cells under OGD/R.

F. Relative mRNA levels of PTRF, as determined by qRT-PCR in the ipsilateral cerebral cortex of the I/R groups treated with echinomycin at the indicated doses (n = 3).

G. Western blot analyses of PTRF in the ipsilateral cerebral cortex of the I/R groups treated with echinomycin at the indicated doses.

H. Relative mRNA levels of PTRF, as determined by qRT-PCR in the ipsilateral cerebral cortex of the I/R groups treated with S3I-201 at the indicated doses (n = 3).

I. Western blot analyses of PTRF in the ipsilateral cerebral cortex of the I/R groups treated with S3I-201 at the indicated doses.

J. qRT-PCR and Western blot analyses of EPO, VEGF, and Glut-1 in the ipsilateral cerebral cortex of the I/R groups treated with echinomycin (10 μg/kg).

K. qRT-PCR and Western blot analyses of VEGF, MMP 9, and cyclin D1 in the ipsilateral cerebral cortex of the I/R groups treated with S3I-201 (5 mg/kg).

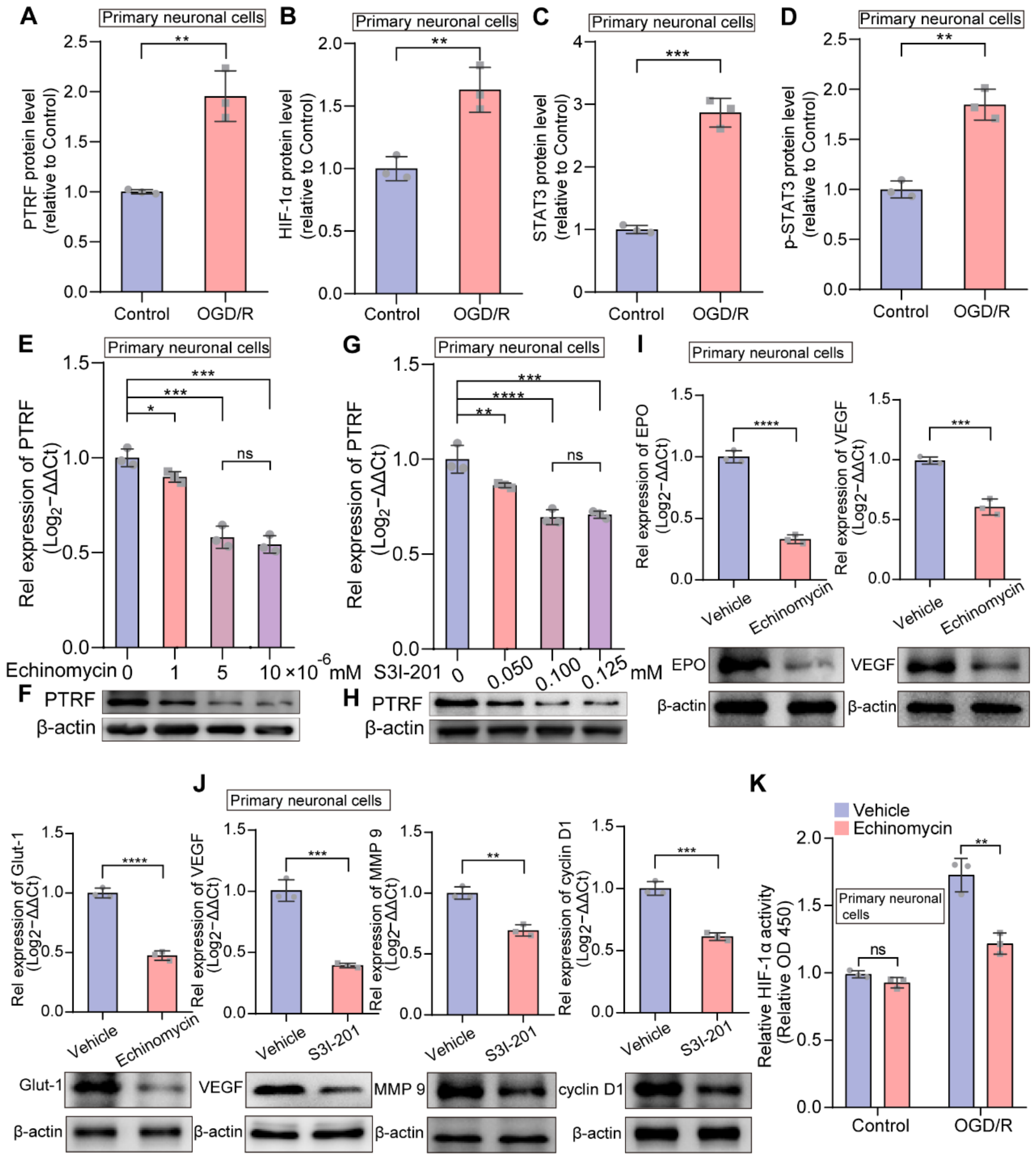

Figure S3. Expression of PTRF is increased in HIF-1α and STAT3-dependent manners in primary neuronal cells under OGD/R.

A-D. Quantification of PTRF, HIF-1α, STAT3, and p-STAT3 proteins normalized to β-actin in primary neuronal cells under OGD/R (n = 3).

E. Relative mRNA level of PTRF, as determined by qRT-PCR in primary neuronal cells treated with echinomycin at the indicated doses under OGD/R (n = 3).

F. Western blot analysis of PTRF in primary neuronal cells treated with echinomycin

at the indicated doses under OGD/R.

G. Relative mRNA level of PTRF, as determined by qRT-PCR in primary neuronal cells treated with S3I-201 at the indicated doses under OGD/R (n = 3).

H. Western blot analysis of PTRF in primary neuronal cells treated with S3I-201 at the indicated doses under OGD/R.

I. qRT-PCR and Western blot analyses of EPO, VEGF, and Glut-1 in primary neuronal cells treated with echinomycin (5×10^{-6} mM) under OGD/R.

J. qRT-PCR and Western blot analyses of VEGF, MMP 9, and cyclin D1 in primary neuronal cells treated with S3I-201 (0.100 mM) under OGD/R.

K. HIF-1α DNA binding activity was measured in primary neuronal cells treated with echinomycin (5×10^{-6} mM) using HIF-1α Transcription Factor Assay Kit (n = 3).

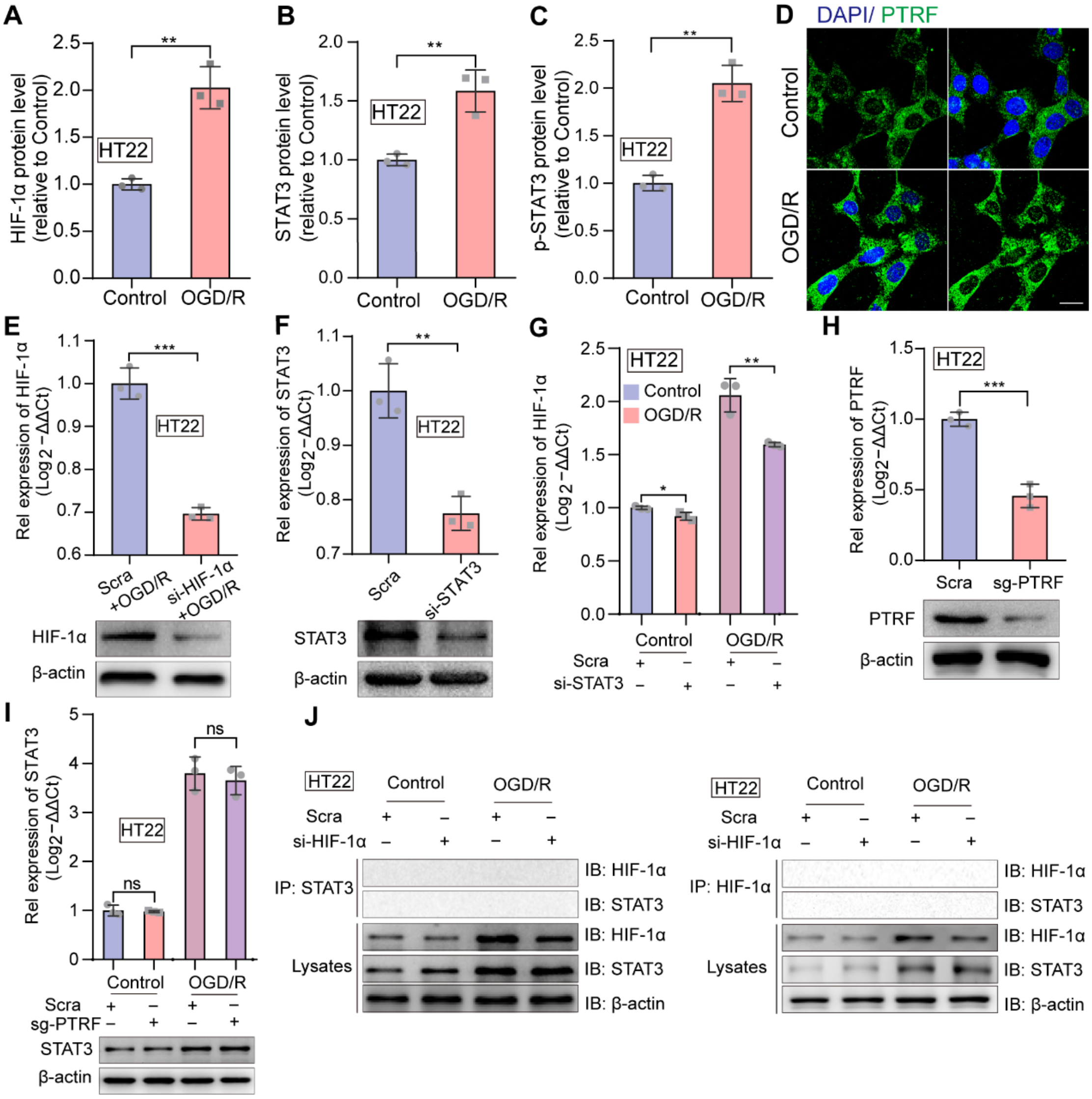

Figure S4. Expression of PTRF is improved in HT22 cells under OGD/R.

A-C. Quantification of HIF-1α, STAT3, and p-STAT3 proteins normalized to β-actin in HT22 cells under OGD/R (n = 3).

D. IF staining of PTRF in HT22 cells under OGD/R. Scale bars = 20 μm.

E-F. mRNA level and Western blot analyses of HIF-1α (E) and STAT3 (F) expression in HT22 cells transfected with HIF-1α and STAT3 siRNA, respectively.

G. mRNA level analysis of HIF-1α expression in HT22 cells transfected with STAT3 siRNA under OGD/R.

H. mRNA level and Western blot analyses of PTRF expression in PTRF KO HT22

cells.

I. mRNA level and Western blot analyses of STAT3 expression in PTRF KO HT22 cells under OGD/R.

J. Cells were lysed from HT22 cells transfected with HIF-1α siRNA or scramble and processed by IP and Western blot with the indicated antibodies.

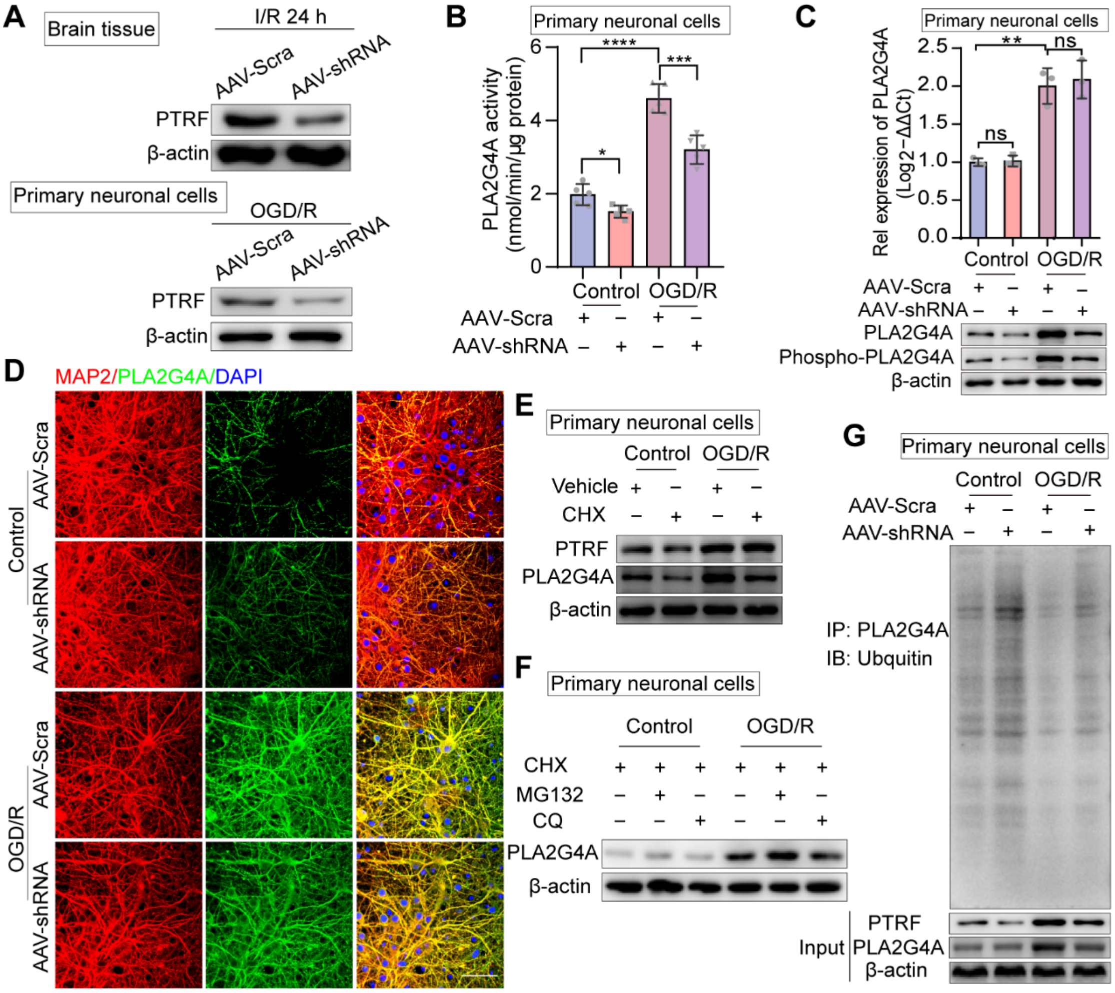

Figure S5. PTRF regulates the activity and stability of PLA2G4A in primary neuronal cells under OGD/R.

A. Representative Western blot analysis of PTRF expression in the ipsilateral cerebral cortex from the mice transfected with AAV-shRNA or AAV-Scramble after cerebral

I/R injury, and in AAV-shRNA or AAV-Scramble transfected primary neuronal cells under OGD/R.

B. PLA2G4A activity in AAV-shRNA or AAV-Scramble transfected primary neuronal cells under OGD/R based on the PLA2G4A assay (n = 5).

C. mRNA level, representative Western blot analyses of PLA2G4A, and Western blot analyses of phospho-PLA2G4A expression in AAV-shRNA or AAV-Scramble transfected primary neuronal cells under OGD/R.

D. IF of PLA2G4A, MAP-2, and nuclei in AAV-shRNA or AAV-Scramble transfected primary neuronal cells under OGD/R. Scale bar = 50 μm.

E. Western blot analysis of PTRF and PLA2G4A in primary neuronal cells treated with CHX (0.050 mM) under OGD/R.

F. Western blot analysis of PLA2G4A in primary neuronal cells after treatment with CHX (0.050 mM), MG132 (0.005 mM) or CQ (0.010 mM).

G. Primary neuronal cells were lysed and subjected to co-IP with an antibody against PLA2G4A and analyzed by Western blot with an anti-ubiquitin antibody.

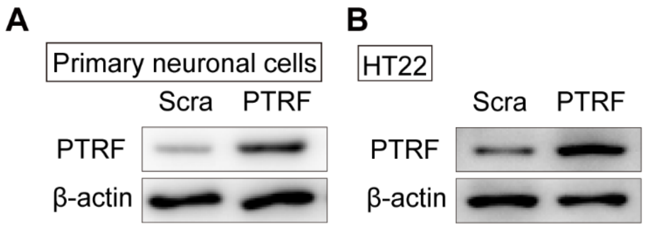

Figure S6. Stable PTRF overexpression in primary neuronal cells and HT22 cells were successfully constructed.

A-B. Western blot analyses of PTRF expression in primary neuronal cells (A) and

HT22 cells (B) transfected with PTRF overexpression lentivirus.

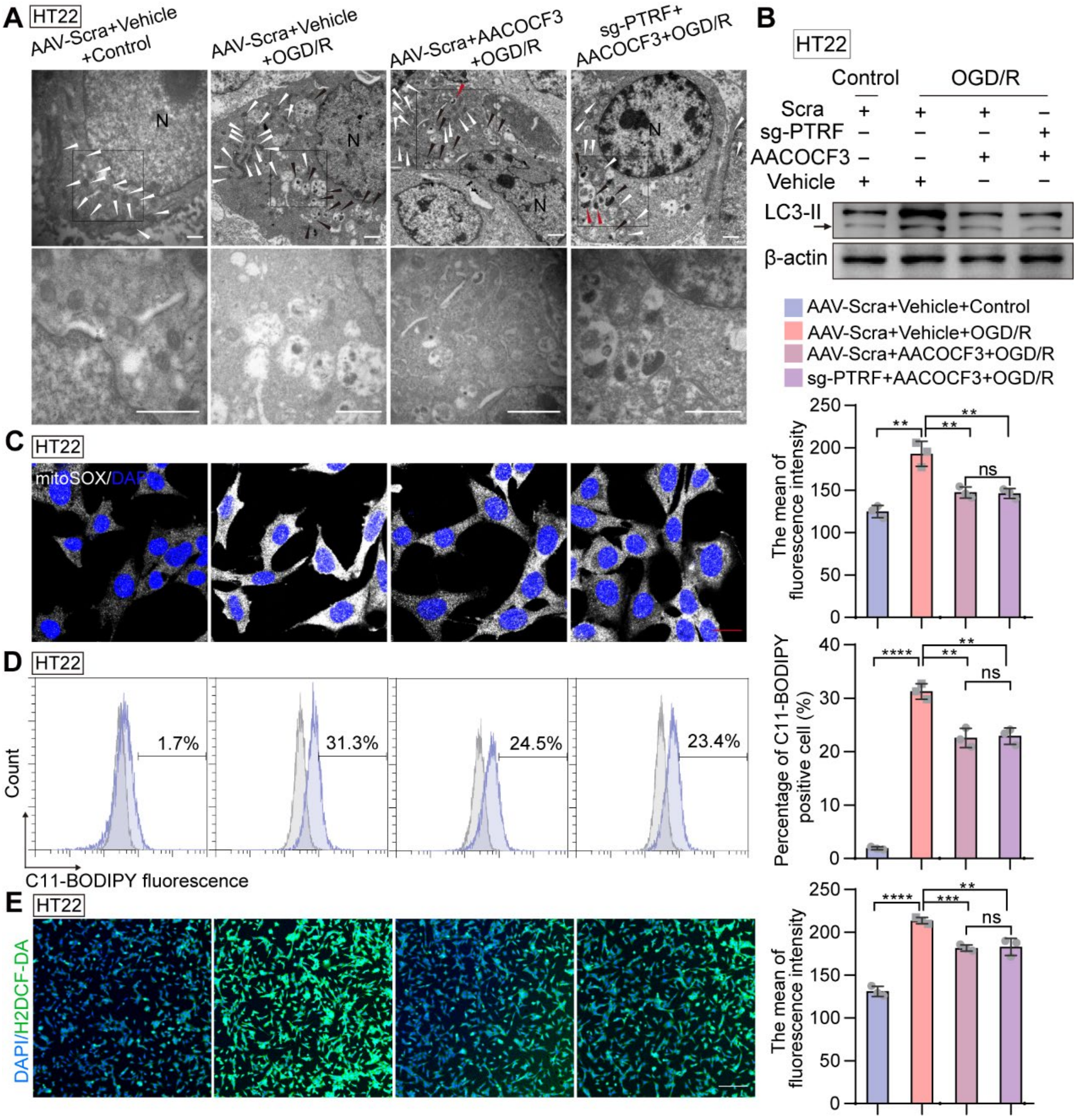

Figure S7. Knockout of PTRF renders HT22 cells blunt to autophagy and decreases lipid peroxidation and ferroptosis via PLA2G4A under OGD/R.

A. TEM of the PTRF KO HT22 cells co-treated with AACOCF3 (0.050 mM) under OGD/R. White arrowheads, mitochondria; black arrowheads, autophagy; red arrowheads, the lysosome. Scale bar = 1 μm.

B. Representative Western blot analysis of the LC3-II levels in PTRF KO HT22 cells

co-treated with AACOCF3 under OGD/R.

C. Representative images of mitoSOX stained PTRF KO HT22 cells co-treated with AACOCF3 under OGD/R. HT22 cells were counterstained with DAPI to visualize cell nuclei. Panel right shows the quantification of the mean fluorescence intensity of mitoSOX (n = 3).

D. Flow cytometry analysis of lipid peroxidation (C11-BODIPY) in PTRF KO HT22 cells co-treated with AACOCF3 under OGD/R. The histogram summarizes the percentage of positive HT22 cells in C11-BODIPY using flow cytometry (n = 3).

E. Representative images of intracellular ROS levels (H2DCF-DA) in PTRF KO HT22 cells co-treated with AACOCF3 under OGD/R. The histograms summarize the mean fluorescence intensity of H2DCF-DA in HT22 cells (n = 3).

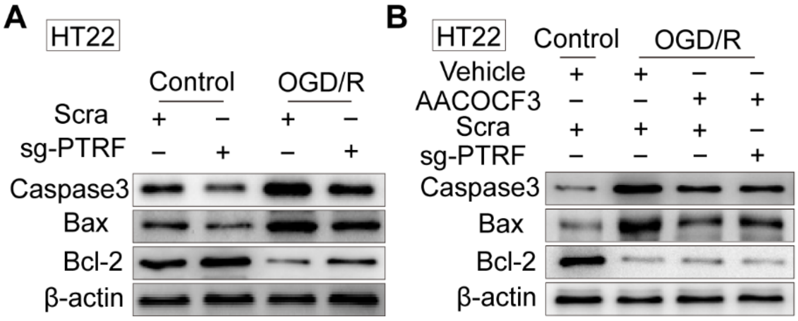

Figure S8. Knockout PTRF in HT22 cells decreases neuronal insults via PLA2G4A under OGD/R.

A. The expression levels of Caspase3, Bax, and Bcl-2 were measured by Western blot analysis in PTRF KO HT22 cells under OGD/R.

B. The expression levels of Caspase3, Bax, and Bcl-2 were measured by Western blot analysis in PTFR KO HT22 cells co-treated with AACOCF3 (0.050 mM) under OGD/R.

Table S1. Detailed description of antibodies used in this study.

Antibodies	Source	Identifier
Rabbit anti-NeuN	Abcam	ab177487
Mouse anti-NeuN	Millipore	MAB337
Rabbit anti-PTRF	proteintech	18892-1-AP
Mouse anti-MAP-2	Immunoway	YM1061
Mouse anti-GFAP	Abcam	ab279289
Mouse anti-Iba-1	Abcam	ab283319
Mouse anti-STAT3	Cell Signaling Technology (CST)	9139S
Rabbit anti-HIF-1α	CST	36169S
Rabbit anti-Phospho-STAT3	CST	4904S
Rabbit anti-β-actin	Abcam	ab8227
Rabbit anti-EPO	Affinity	AF5190
Rabbit anti-VEGF	Affinity	AF5131
Rabbit anti-Glut-1	Immunoway	YT1928
Rabbit anti-cyclin D1	Immunoway	YT1172
Rabbit anti-MMP 9	Affinity	AF5228
Rabbit anti-Histone H3	CST	4499S
Rabbit anti-CBP	CST	7389S
Rabbit anti-PLA2G4A	CST	5249S
Rabbit anti-Phospho- PLA2G4A	CST	53044S
Mouse anti-Ubiquitin	CST	3936S

Mouse anti-LC3	Novus Biologicals	NB100-2220
Mouse anti -caspase3	Affinity	AF6311
Mouse anti-Bax	Affinity	AF0120
Mouse anti-Bcl2	Affinity	AF6139
Alexa Fluor^{TM} 594 phalloidin	invitrogen	1906348
Alexa Fluor^{TM} 633 goat anti-mouse IgG (H+L)	invitrogen	A21052
Alexa Fluor^{TM} 594 goat anti-mouse IgG (H+L)	invitrogen	A11032
Alexa Fluor^{TM} 488 donkey anti-mouse IgG (H+L)	invitrogen	A21202
Alexa Fluor^{TM} 488 goat anti-rabbit IgG (H+L)	invitrogen	A11008
Alexa Fluor^{TM} 594 donkey anti-rabbit IgG (H+L)	invitrogen	A21207
Alexa Fluor^{TM} 594 donkey anti-goat IgG (H+L)	invitrogen	A11058

Table S2. The primers used for qRT-PCR.

Target		Sequence
β-actin mRNA	Forward	TGCACTGTGCGGCGAGGC
	Reverse	TCGAGCCATAAAAGGCAA
PTRF mRNA	Forward	TGAATCAGTGTCTGACCGCC
	Reverse	CCCGAGCACTGCAGAATACA
EPO mRNA	Forward	CACCCTGCTGCTTTTACTCT
	Reverse	AACCCATCGTGACATTTTCT
VEGF mRNA	Forward	ACCGCGAGGCAGCTTGAGTTA
	Reverse	ACCGCCTTGGCTTGTCACAT
Glut-1 mRNA	Forward	AGCCCTGCTACAGTGTATCCT
	Reverse	CCGACCCTCTTCTTTCATCT
cyclin D1 mRNA	Forward	GGATGCTGGAGGTCTGTGAG
	Reverse	CACAACTTCTCGGCAGTCAA
MMP 9 mRNA	Forward	GTCATTCGCGTGGATAAGGA
	Reverse	AGGCTTTGTCTTGGTACTGG
hif1-alpha mRNA	Forward	AACTGCCACCACTGATGAAT
	Reverse	CCACTGTATGCTGATGCCTTA
STAT3 mRNA	Forward	TTCAGCGAGAGCAGCAAAGA
	Reverse	ACTTGGTCTTCAGGTACGGG
PLA2G4A mRNA	Forward	GTGAGGGGCTTTATTCCACA
	Reverse	GGTGAGAGTACAAGGTTGACA

Table S3. The sequences of siRNAs targeting HIF-1α or STAT3.

Target	Catalog	Sequence
siRNA (scramble)	siRNA#	5’ –TTCTCCGAACGTGTCACGTTT –3’
mus-hif1-alpha	siRNA#	5’ –CAGAGACGAAGGACAATAAAG–3’
mus-Stat3	siRNA#	5’ –GAGUUGAAUUAUCAGCUUAAA–3

Table S4. The primers used for ChIP.

Target		Sequence
transcription factor hif1-alpha		
PTRF primer 1	Forward	GGGCAGAAGAGAGGACATCG
	Reverse	TATAACCATCACAATGGGCAGA
PTRF primer 2	Forward	ATAGACATCTGCCTGTCTGCC
	Reverse	TTGAGGTTAAAGAAGAGAGACCCT
PTRF primer 3	Forward	AGTACCGCTATAGTTGCCCC
	Reverse	GATCACGGAACCCCATCTCT
transcription factor STAT3		
PTRF primer 1	Forward	ATAGACATCTGCCTGTCTGCC
	Reverse	TTGAGGTTAAAGAAGAGAGACCCT
PTRF primer 2	Forward	TGGGCACCAGACTAGAAGTG
	Reverse	GACACAGGCCCTCAGCAATC
PTRF primer 3	Forward	CCCAAGGGAAGAACGAGTCC
	Reverse	TGAGTTACCACTGTCCCCCA
PTRF primer 4	Forward	CGCCTTAATCATGCCCTGTC
	Reverse	GCGCGTGAATTGGGTGTAAA
